# Statin action favors normalization of the plasma lipidome in the atherogenic mixed dyslipidemia of MetS: potential relevance to statin-associated dysglycemia[S]

**DOI:** 10.1194/jlr.P061143

**Published:** 2015-12

**Authors:** Peter J. Meikle, Gerard Wong, Ricardo Tan, Philippe Giral, Paul Robillard, Alexina Orsoni, Neil Hounslow, Dianna J. Magliano, Jonathan E. Shaw, Joanne E. Curran, John Blangero, Bronwyn A. Kingwell, M. John Chapman

**Affiliations:** Baker IDI Heart and Diabetes Institute,* Melbourne, Australia; Dyslipidemia and Atherosclerosis Research Unit,† INSERM UMR-S939, and University of Pierre and Marie Curie, Pitie-Salpetriere University Hospital, Paris, France; Kowa Research Europe Ltd.,§ Wokingham, United Kingdom; South Texas Diabetes and Obesity Institute,** University of Texas Health Science Center at San Antonio, Harlingen, TX

**Keywords:** lipidomics, obesity, plasmalogens, cholesterol, omega-3 fatty acids, pitavastatin, metabolic syndrome

## Abstract

The impact of statin treatment on the abnormal plasma lipidome of mixed dyslipidemic patients with metabolic syndrome (MetS), a group at increased risk of developing diabetes, was evaluated. Insulin-resistant hypertriglyceridemic hypertensive obese males (n = 12) displaying MetS were treated with pitavastatin (4 mg/day) for 180 days; healthy normolipidemic age-matched nonobese males (n = 12) acted as controls. Statin treatment substantially normalized triglyceride (−41%), remnant cholesterol (−55%), and LDL-cholesterol (−39%), with minor effect on HDL-cholesterol (+4%). Lipidomic analysis, normalized to nonHDL-cholesterol in order to probe statin-induced differences in molecular composition independently of reduction in plasma cholesterol, revealed increment in 132 of 138 lipid species that were subnormal at baseline and significantly shifted toward the control group on statin treatment. Increment in alkyl- and alkenylphospholipids (plasmalogens) was prominent, and consistent with significant statin-induced increase in plasma polyunsaturated fatty acid levels. Comparison of the statin-mediated lipidomic changes in MetS with the abnormal plasma lipidomic profile characteristic of prediabetes and T2D in the Australian Diabetes, Obesity, and Lifestyle Study and San Antonio Family Heart Study cohorts by hypergeometric analysis revealed a significant shift toward the lipid profile of controls, indicative of a marked trend toward a normolipidemic phenotype. Pitavastatin attenuated the abnormal plasma lipidome of MetS patients typical of prediabetes and T2D.

The HMG-CoA reductase inhibitors, or statins, act to stabilize vulnerable lipid-rich atherosclerotic plaques and to reduce cardiovascular morbidity and mortality as a consequence of their marked lowering of circulating levels of atherogenic apoB-containing lipoproteins, and notably of LDL-cholesterol (LDL-C) (1–3). Moreover, statin therapy is well tolerated, with a low incidence of associated adverse events (1, 2, 4). Nonetheless, adverse effects are of clinical significance; among such effects is a potential increased risk of development of new-onset T2D over the long term (5). Importantly, post hoc analyses of the Treating to New Targets (TNT), IDEAL, and JUPITER trials indicate that subjects with preexisting risk factors for development of T2D, such as hyperglycemia, hypertriglyceridemia, hypertension, and elevated BMI, are more susceptible to statin-induced T2D than those without, thereby suggesting a predisposition (5, 6). Each of these risk factors is a component of the cluster characteristic of the metabolic syndrome (MetS), a prediabetic state characterized by elevated cardiovascular risk (7); atherogenic mixed dyslipidemia, characterized by elevated levels of triglyceride-rich lipoproteins and subnormal concentrations of HDL-cholesterol (HDL-C), contributes significantly to such risk (8, 9). Furthermore, the recent findings in the population-based Metabolic Syndrome in Men (METSIM) cohort revealed that statin-treated subjects encountered a 46% increased risk of T2D; two mechanisms were principally involved, i.e., decrease in both insulin sensitivity and secretion (10). In addition, inhibition of the target enzyme, HMG-CoA reductase, may equally impact the putative actions of statins on glucose homeostasis by indirect mechanisms (11).

The potential dysglycemic effect of statins, based on available evidence, appears to represent a class effect, although individual statins at different doses were found to display differing potential to increase the incidence of T2D in recent meta-analyses (12–15). The cholesterol-lowering effect of statins is exerted via the inhibition of cholesterol biosynthesis, principally in the liver, with upregulation of LDL receptors; enhanced catabolism of LDL and other apoB-containing lipoproteins ensues, with a decrease in their plasma levels (3). Reduction in atherogenic particles is equally associated with changes in lipoprotein composition, some of which result from a decrease in CETP activity (16). As 95% or more of plasma lipids are transported in lipoproteins, which facilitate exchange and net transfer of lipids with cells of the circulation, tissues, and organs, then the complement of individual lipid molecular species in plasma, i.e., the lipidome, has the potential to reflect chronic disorders of lipid metabolism, such as those implicated in prediabetes and T2D.

Comparison of the plasma lipidome in large cohorts of healthy, prediabetic, and T2D individuals displaying mixed dyslipidemia with those of normal glucose-tolerant normolipidemic subjects by mass spectrometric analysis has identified significant associations between specific plasma lipid classes and insulin-resistant states, i.e., prediabetes and T2D (17, 18). Circulating levels of ceramide, phosphatidylinositol, phosphatidylethanolamine, and phosphatidylglycerol were positively associated with prediabetes and T2D after adjustment for age, sex, and obesity, while alkyl- and alkenylphosphatidylcholines (i.e., plasmalogens) were negatively associated (17). Several of these lipid classes have been mechanistically linked to the development of insulin resistance, oxidative stress, and inflammation, thereby providing new insight into the mechanisms underlying the perturbed lipid metabolism associated not only with prediabetic and diabetic states, but also with atherosclerotic coronary disease (17–22). Indeed, such perturbed lipid metabolism in insulin-resistant states is typically manifested as a mixed dyslipidemia, and may indeed be causal in the development of insulin resistance (9).

We hypothesize that statin treatment may impact the risk of development of T2D in patients with atherogenic mixed dyslipidemia and MetS [defined according to strict International Diabetes Federation (IDF) criteria] through modulation of biologically active plasma lipids relevant to the pathogenesis of diabetes. In the CAPITAIN trial, an open label study, we evaluated this hypothesis through analysis of the plasma lipidome in male patients with the above phenotype at baseline and after pitavastatin (4 mg/day) treatment for a period of 6 months; subjects acted as their own controls. Modifications in the plasma lipidome resulting from statin therapy were then compared with the corresponding profiles associated with T2D in two large cohorts, i.e., the Australian Diabetes, Obesity, and Lifestyle Study (AusDiab) and the San Antonio Family Heart Study (SAFHS) cohorts (17). In this way, we evaluated the degree to which the plasma concentrations of key bioactive lipids associated with diabetes in dyslipidemic MetS patients were modified, either toward the lipidomic profile of control nondiabetic nondyslipidemic subjects, or in contrast, toward the diabetic lipidomic profile by statin treatment.

## MATERIALS AND METHODS

### The CAPITAIN trial

The CAPITAIN trial was an open label study of the chronic and acute effects of pitavastatin on monocyte phenotype, endothelial dysfunction, and HDL atheroprotective function in subjects with MetS.

### Patient cohort

**Fig. 1.**
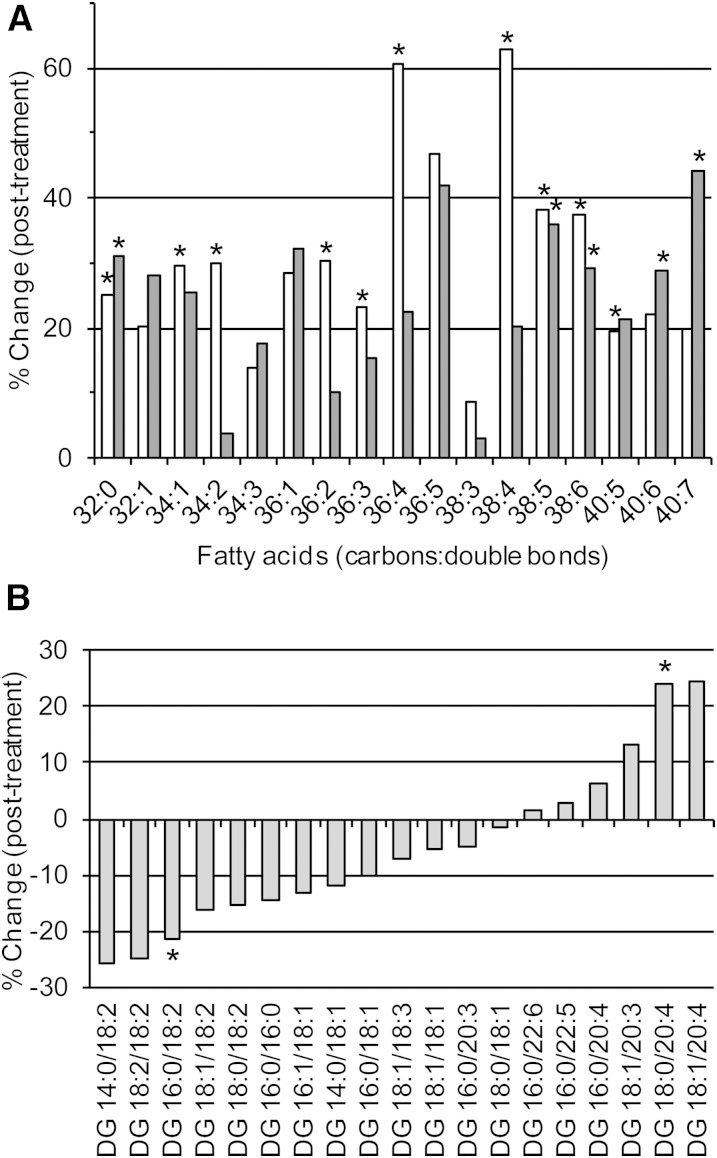
Effect of pitavastatin treatment (4 mg/day) on plasma phosphatidylcholine, phosphatidylethanolamine, and diacylglycerol (DG) species (normalized to nonHDL-C) between MetS subjects pretreatment (D0) and MetS subjects posttreatment (D180). Plasma lipids were analyzed as described in the Materials and Methods and normalized to nonHDL-C. The mean percent change from MetS pretreatment (D0) to MetS posttreatment (D180) was calculated and the significance determined using a paired Student’s *t*-test correcting for multiple comparisons by the Benjamini-Hochberg method. A: Shows phosphatidylcholine species (light bars) and phosphatidylethanolamine species (dark bars). B: Shows diacylglycerol species. *Indicates corrected *P* value <0.05.

The CAPITAIN study (ClinicalTrials.gov: NCT01595828) (23) was monocentric and recruited 12 Caucasian male subjects aged 30–65 years (mean age 50 ± 3 years) who were nonsmokers for at least 12 months and who had smoked less than 25 cigarettes per day on a regular basis, with plasma LDL-C of 130–190 mg/dl (3.4–4.9 mmol/l) and a diagnosis of MetS according to strict IDF criteria (7). All subjects displayed atherogenic mixed dyslipidemia with plasma triglycerides >150 mg/dl (1.7 mmol/l) (see Fig. 1); participants were required to have central obesity (defined as a waist circumference ≥94 cm), plus any two of: *i*) triglycerides ≥1.7 mmol/l, HDL-C <1.0 mmol/l; *ii*) controlled hypertension [systolic blood pressure (SBP) ≥130 mmHg or diastolic blood pressure ≥85 mmHg] or treatment for previously diagnosed hypertension with a calcium channel blocker who did not require treatment with a diuretic, β-blocker, angiotensin converting enzyme inhibitor, or angiotensin II receptor blocker; and/or *iii*) fasting plasma glucose (FPG) ≥5.6 mmol/l. Plasma samples from overnight-fasted healthy control male subjects (n = 12) were retrieved from the Baker IDI biobank; these nonobese subjects were consuming a Western-type diet commensurate with their BMI (mean 23.1 ± 2.5 kg/m^2^). Equally, these subjects were matched for age (49 ± 11 years), but were not hypertensive (SBP = 119 ± 10 mmHg), hyperglycemic (FPG = 5.0 ± 0.7 mmol/l), or dyslipidemic (Table 1); furthermore, they had no history of cardiovascular disease or T2D.

The plasma lipid profiles of the participants and control subjects are summarized in Table 1. Detailed baseline parameters of glucose homeostasis and insulin resistance in participants in the CAPITAIN trial were reported earlier (23). Key exclusion criteria were fasting triglyceride levels >400 mg/dl (4.5 mmol/l), LDL-C >190 mg/dl (4.9 mmol/l), and excessive obesity defined as BMI >35 kg/m^2^. A detailed listing of exclusion criteria is given in the supplementary data.

### Study design

All participants in the CAPITAIN trial underwent screening within 3 weeks of inclusion and study drug administration. Participants were treated with pitavastatin (4 mg/day) for 180 days; with the exception of study visits, the study drug was taken in the morning between 7:00 and 10:00 AM. To initiate the protocol, each subject was admitted to the Clinical Unit at approximately 6:00 PM on day −2, and remained until baseline blood collection in the overnight fasting state on day 1. Thus, all subjects remained for 36 h in the Clinical Unit before blood sampling in order to ensure abstinence from alcohol, coffee, tea, or sugared beverages; meals consumed on day −2 and on day −1 prior to collection of the pretreatment (D0) blood sample were mixed meals consisting of 30–35% fat, 50–55% carbohydrate, and approximately 15% protein. Strenuous physical exercise was not allowed during the stay in the Clinical Unit. Subjects took the study medication at the end of the visit. All subjects were counseled by a dietician to abstain from alcohol, coffee, tea, or sugared beverages, or any beverages containing methylxanthines (theophylline, caffeine, or theobromine) during the 48 h preceding this and subsequent visits. Furthermore, subjects were requested to limit, as much as possible, the consumption of all of the former beverages throughout the study duration. The consumption of starfruit, grapefruit, or grapefruit juice was not allowed starting from 1 week before dosing until discharge at the final visit. Otherwise, overall dietary intake was not modified during the study. Subjects returned to the Clinical Unit on days 7, 30, 42, and 120 for compliance (drug intake was monitored with individual diary cards and by pill counts) and safety assessments; the investigator checked on the well-being of all subjects prior to discharge at each visit. At the end of each visit, the participant took the study medication. Finally, subjects returned to the Clinical Unit on day 180 (±7 days) in the morning, having fasted for at least 12 h, for blood sample collection and compliance and safety assessments. The last meal taken before the study visit corresponded to a mixed meal, as described above and as counseled by the dietician. Blood samples were withdrawn in the Clinical Unit by venipuncture from the cubital vein into precooled (4°C) EDTA-containing tubes (final concentration 1 mg/ml) at pretreatment (D0) and posttreatment (D180) time points; plasma was separated by centrifugation at 1,700 *g* for 15 min at 4°C, and was stored at −80°C after the addition of 0.05% sucrose. Plasma samples were aliquoted and frozen at −80°C within 2 h of blood collection; earlier studies have documented the absence of lipid- or protein-derived oxidation products in the component lipoproteins of such samples (24, 25).

All participants gave written informed consent after the purpose and nature of the investigation had been explained to them. The study was approved by the Institutional Review Boards or Ethics Committees of the participating centers and was conducted according to the principles of the Declaration of Helsinki (2013).

### Sample preparation and lipid extraction

The order of the plasma samples was randomized prior to lipid extraction and analysis. Whole plasma samples were analyzed in triplicate and the average values taken for subsequent statistical analyses. Quality control plasma samples were included at a ratio of 1:12. Total lipid extraction from a 10 μl aliquot of plasma was performed by a single phase chloroform:methanol (2:1) extraction, as described previously (26).

### Lipidomic analysis

Lipid analysis was performed by liquid chromatography-electrospray ionization-tandem mass spectrometry using an Agilent 1200 liquid chromatography system combined with an Applied Biosystems API 4000 Q/TRAP mass spectrometer with a turbo-ionspray source (350°C) and Analyst 1.5 data system. Lipid species (330 in total) from the following lipid classes and subclasses were analyzed: dihydroceramide (dhCer), ceramide (Cer), monohexosylceramide (MHC), dihexosylceramide (DHC), trihexosylcermide (THC), G_M3_ ganglioside (GM3), SM, phosphatidylcholine (PC), alkylphosphatidylcholine [PC(O)], alkenylphosphatidylcholine [plasmalogen, PC(P)], lysophosphatidylcholine (LPC), lysoalkylphosphatidylcholine [lysoplatelet activating factor, LPC(O)], phosphatidylethanolamine (PE), phosphatidylinositol (PI), phosphatidylserine (PS), phosphatidylglycerol (PG), cholesteryl ester (CE), free cholesterol (COH), diacylglycerol (DG), and triacylglycerol (TG) (Table 2, supplementary Table 1) (26). The abbreviations shown above are only used when referring to individual lipid species, as in LPC 22:6, which define a lysophosphatidylcholine with a fatty acid containing 22 carbons and six double bonds. For a number of the lipids which contain two fatty acid chains, the mass spectrometry-based measurements here do not directly determine the constituent fatty acids; rather the sum of the number of carbons and the sum of the number of double bonds across both fatty acids is determined. Accordingly, we denote these species by the combined length and number of double bonds, e.g., PC 36:4.

Relative amounts of each molecular lipid species were calculated by expressing the peak area of each species relative to the peak area of the corresponding stable isotope or nonphysiological internal standard, as described previously (26). Concentrations of total lipid classes were calculated from the sum of the individual lipid species within each class. The methodology, instrumentation, and internal standards used were identical to our previous studies to characterize the associations between plasma lipids and T2D (17).

Whole plasma was analyzed in triplicate and the mean values of each triplicate subsequently used for statistical analysis. Assay performance was monitored by calculating the coefficient of variation (percent) of the quality control plasma samples across the entire analytical run. Across the 330 lipid species, the precision of the extraction and analytical process had a median coefficient of variation of 7.0% and an interquartile range of 5.0–10.8%.

### Statistical analysis

Plasma lipid concentrations were analyzed either directly or after normalization to nonHDL-C (defined as total cholesterol minus HDL-C). Mean percent differences between groups [control, MetS pretreatment (D0), and MetS posttreatment (D180)] were calculated and significance determined using Student’s *t*-test. *P* values were corrected for multiple comparisons using the Benjamini-Hochberg method.

In order to assess the effect of pitavastatin on the diabetogenic lipid profile (i.e., those lipids previously established to be associated with incident T2D) at baseline in CAPITAIN, we aligned those lipids that were significantly modified following pitavastatin treatment with the lipids significantly associated with T2D (17). These associations were previously determined in a subset of the AusDiab study (n = 351) (27) and had been validated in the larger SAFHS (n = 1,076) (28). It is noteworthy that plasma samples were taken in the overnight-fasted state in AusDiab participants, who were consuming Western-type diets commensurate with their BMI values (median 27.9, range 25.5–30.7) (17).

To align the analytical methodologies between studies, lipid concentrations in both the diabetic cohort studies and in the CAPITAIN study were normalized to nonHDL-C and the associations with T2D in the AusDiab study and SAFHS were recalculated. Normalization to nonHDL-C adjusted for the major effect of pitavastatin in reducing atherogenic cholesterol levels and allowed us to assess modifications in the plasma lipidome that were independent of the primary change in nonHDL-C. Hypergeometric tests were applied to assess the significance of the overlap (i.e., similarity) between the set of lipids negatively associated with T2D/prediabetes with the lipid set which was positively regulated by pitavastatin treatment (a potential anti-diabetogenic effect). The significance of the overlap between the set of lipids positively associated with T2D/prediabetes and the lipid set which was positively regulated by pitavastatin treatment (a potential pro-diabetogenic effect) was equally determined.

Network analysis was performed to identify clusters of correlated lipid species that were significantly associated with T2D in the AusDiab study. The change in these lipids resulting from pitavastatin treatment in the CAPITAIN study was then determined. Pearson’s linear correlation was computed between pairs of lipids based on all subjects from the AusDiab cohort. Edges were defined if the correlation between node pairs (lipids) was statistically significant (Benjamini-Hochberg corrected *P* value ≤0.05). Subsequent correlation analyses of the changes induced by pitavastatin treatment were also mapped onto the network to identify responses to pitavastatin treatment that were associated with T2D.

## RESULTS

### Anthropometric, blood pressure, plasma lipid, and glucose values in MetS versus control subjects

The MetS group, composed of 12 phenotyped Caucasian adult males, was hypertriglyceridemic (baseline triacylglycerol level, 216 mg/dl, 2.4 mmol/l; as compared with 75 mg/dl, 0.85 mmol/l in the control group; *P* = 3.8 × 10^−8^ vs. controls) and exhibited a significantly lower HDL-C concentration (46.3 mg/dl, 1.19 mmol/l) relative to the control group (56.8 mg/dl, 1.46 mmol/l; *P* = 1.7 × 10^−3^). Interestingly, our previous study in MetS subjects (n = 10) revealed a similar mean baseline HDL-C level of 47 mg/dl, which was again significantly lower than that of the control group (57 mg/dl) (29). Clearly then, HDL-C levels in our MetS cohort were subnormal, but superior to the cutoff value of 40 mg/dl (1 mmol/l) proposed by IDF for males with MetS. Plasma total cholesterol (*P* = 6.5 × 10^−3^), LDL-C (*P* = 1.2 × 10^−4^), and nonHDL-C (*P* = 1.0 × 10^−3^) levels were higher in the MetS group relative to control subjects, indicative of their hypercholesterolemia (**Table 1**). Consistent with inclusion criteria for MetS, CAPITAIN subjects displayed a higher BMI (31.7 ± 1.6 kg/m^2^ vs. 23.1 ± 2.5 kg/m^2^, *P* = 1.2 × 10^−9^), together with elevated SBP (131 ± 11 mmHg vs. 119 ± 10 mmHg, *P* = 8.2 × 10^−3^) and FPG (94.7 ± 7.2 mg/dl vs. 84.7 ± 12.6 mg/dl; 5.2 ± 0.4 mmol/l vs. 5.0 ± 0.7 mmol/l, nonsignificant) relative to controls. As previously reported, this group had a homeostasis model of insulin resistance (HOMA-IR) score of 2.7 ± 1.7 (standard deviation) (23), placing them as a group in the insulin-resistant range (30). Baseline low levels of systemic inflammation, as determined by high-sensitivity C-reactive protein (1.6 ± 0.2 mg/l), did not change on statin treatment.

**TABLE 1.** Characteristics of dyslipidemic male participants with MetS and of the effect of statin treatment for 180 days in the CAPITAIN study compared with the healthy control male group

Characteristic	Healthy Control*^a^*	Baseline (D0)*^a^*	Follow-up (D180)*^a^*	*P* [Baseline (D0) vs. Healthy Control]*^b^*	*P* [Follow-up (D180) vs. Healthy Control]*^b^*	*P* [Follow-up (D180) vs. Baseline (D0)]*^c^*
Age	49 ± 11	50 ± 12	50 ± 12	n.s.	n.s.	n.s.
BMI (kg/m^2^)	23.1 ± 2.5	31.7 ± 1.6	31.8 ± 2.2	**1.17E-09**	**7.35E-09**	n.s.
FPG (mmol/l)	4.96 ± 0.7	5.17 ± 0.4	5.38 ± 0.46	n.s.	**3.86E-02**	n.s.
HOMA-IR	n.d.	2.7 ± 1.7	2.2 ± 0.9	**—**	—	n.s.
Cholesterol (mmol/l)	4.44 ± 0.88	6.01 ± 1.57	4.18 ± 0.51	**6.51E-03**	n.s.	**3.98E-04**
HDL-C (mmol/l)	1.46 ± 0.24	1.2 ± 0.25	1.25 ± 0.32	**1.73E-02**	n.s.	n.s.
NonHDL-C (mmol/l)	2.98 ± 0.89	4.81 ± 1.41	2.94 ± 0.4	**1.02E-03**	n.s.	**5.98E-04**
LDL-C (mmol/l)	2.60 ± 0.84	3.96 ± 0.55	2.48 ± 0.52	**1.18E-04**	n.s.	**1.56E-05**
Triglyceride (mmol/l)	0.85 ± 0.25	2.24 ± 0.53	1.39 ± 0.32	**3.79E-08**	**1.47E-04**	**2.45E-04**

a Values represent mean ± standard deviation. n.d., not determined.

b *P* values determined from unpaired Student’s *t*-test. Bold indicates significant values. n.s., not significant.

c *P* values determined from paired Student’s *t*-test.

### Effect of statin treatment on the plasma lipid profile

LDL-C, nonHDL-C, and remnant cholesterol levels (calculated as total plasma cholesterol − LDL-C − HDL-C, and corresponding primarily to the cholesterol content of triglyceride-rich VLDLs and their remnants in the fasting state) were markedly reduced by pitavastatin treatment [−39% (*P* = 1.6 × 10^−5^), −37% (*P* = 6.0 × 10^−4^), and −38% (*P* = 2.7 × 10^−4^), respectively], such that on-treatment levels did not differ significantly from controls; while triglyceride levels decreased markedly (−38%, *P* = 2.5 × 10^−4^), they remained significantly higher than controls (123.0 mg/dl, 1.39 mmol/l vs. 75.2 mg/dl, 0.85 mmol/l). Levels of HDL-C showed a nonsignificant elevation of 4% on treatment (Table 1). Variable, small increments in HDL-C levels are typical for other members of the statin class, although such effects appear to be unrelated to reductions in nonHDL-C and are largely statin- and dose-dependent (31). apoE genotypes were primarily E3/E3 (n = 9), while two subjects were E3/E4 heterozygotes and one E2/E3.

Finally, no significant changes from baseline in HbA1c, insulin, HOMA-IR, and the quantitative insulin sensitivity check index (QUICKI) were observed in CAPITAIN subjects at D180, although a trend to a decrease in both insulin levels and HOMA-IR was observed on statin treatment (mean baseline insulin at D0, 70.1 pmol/l vs. 55.9 pmol/l at D180; HOMA-IR, mean baseline 2.7 vs. 2.2 at D180). A minor, but significant, increase in FPG (3.8%; *P* < 0.04) at D180 compared with the D0 baseline level (5.38 mmol/l vs. 5.17 mmol/l) was however noted (Table 1), as reported earlier (23). Such effects were independent of the efficacy of pitavastatin in reducing atherogenic lipid levels.

### Comparison of plasma lipidomic profiles in control and MetS subjects and impact of statin treatment

As expected, the plasma lipidome of the MetS group at baseline displayed significantly higher absolute levels of cholesteryl esters (29%, *P* = 6.0 × 10^−3^), diacyl- and triacylglycerols (227%, *P* = 2.5 × 10^−5^ and 171%, *P* = 2.5 × 10^−5^, respectively) in addition to phosphatidylglycerol (67%, *P* = 8.1 × 10^−3^; **Table 2**) relative to the control group. By contrast, the MetS group had significantly lower baseline levels of lysophosphatidylcholine (−17.6%, *P* = 3.4 × 10^−2^), alkenylphosphatidylcholine (plasmalogen, −27.8%, *P* = 6.7 × 10^−3^), as well as alkylphosphatidylethanolamine (−30.5%, *P* = 3.4 × 10^−2^) and alkenylphosphatidylethanolamine (−30.5%, *P* = 2.2 × 10^−2^).

**TABLE 2.** Differences between absolute plasma levels of lipid classes in control subjects, MetS subjects at pretreatment baseline (D0), and MetS subjects post-pitavastatin treatment (D180)

Lipid Class	Relative Concentration (μM)*^a^*	MetS Pretreatment (D0) versus Control		MetS Posttreatment (D180) versus MetS Pretreatment (D0)		MetS Posttreatment (D180) versus Control	
		Mean Percent Difference*^b^*	*P^c^*	Mean Percent Difference*^d^*	*P^c^*	Mean Percent Difference*^e^*	*P^c^*
Dihydroceramide	0.68	15.7	1.59E-01	−17.2	**1.84E-03**	−4.3	7.75E-01
Ceramide	5.0	4.3	7.63E-01	−17.4	**1.88E-03**	−13.8	2.09E-01
Monohexosylceramide	7.5	−4.4	7.63E-01	−30.3	**1.15E-04**	−33.4	**1.09E-03**
Dihexosylceramide	5.8	−11.2	1.13E-01	−25.2	**4.65E-05**	−33.6	**1.77E-05**
Trihexosylceramide	1.8	−17.2	5.59E-02	−24.4	**1.19E-04**	−37.4	**1.26E-04**
GM3 ganglioside	3.0	−13.6	6.57E-02	−22.3	**1.35E-05**	−32.8	**1.26E-04**
Sphingomyelin	322	−0.2	9.77E-01	−18.5	**1.22E-04**	−18.6	**8.85E-03**
Phosphatidylcholine	1471	−0.9	9.05E-01	−14.7	**2.03E-03**	−15.4	**2.82E-03**
Alkylphosphatidylcholine	54	−14.4	1.59E-01	−11.5	**4.14E-02**	−24.2	**8.35E-03**
Alkenylphosphatidylcholine	33	−27.8	**6.65E-03**	−11.5	6.73E-02	−36.1	**1.38E-04**
Lysophosphatidylcholine	194	−17.6	**3.42E-02**	−19.2	**6.81E-03**	−33.4	**1.26E-04**
Lysoalkylphosphatidylcholine	1.1	−18.5	5.59E-02	−23.9	**2.07E-03**	−38.0	**4.60E-05**
Phosphatidylethanolamine	26	31.1	1.79E-01	−22.4	**2.91E-02**	1.7	9.29E-01
Alkylphosphatidylethanolamine	2.9	−30.5	**3.42E-02**	−0.6	9.63E-01	−30.9	**3.96E-02**
Alkenylphosphatidylethanolamine	37	−30.5	**2.23E-02**	−9.6	2.56E-01	−37.2	**2.64E-03**
Lysophosphatidylethanolamine	16	−17.7	8.70E-02	−12.1	1.65E-01	−27.7	**5.96E-03**
Phosphatidylinositol	92	1.0	9.41E-01	−27.7	**4.52E-03**	−27.0	**8.09E-03**
Lysophosphatidylinositol	3.3	−21.7	**9.15E-03**	−1.5	9.11E-01	−22.9	**1.65E-02**
Phosphatidylserine	2.8	0.0	2.69E-01	−46.3	1.33E-01	−60.6	**9.63E-04**
Phosphatidylglycerol	0.12	66.6	**8.11E-03**	−36.0	**6.93E-04**	6.5	7.78E-01
Bis(monoacylglycero)phosphate	0.017	3.9	7.63E-01	0.6	9.63E-01	4.6	7.63E-01
Free cholesterol	873	6.4	5.49E-01	−30.1	**1.44E-05**	−25.6	**4.83E-03**
Cholesteryl ester	1106	29.0	**6.02E-03**	−22.9	**2.03E-03**	−0.5	9.29E-01
Diacylglycerol	23	227.0	**2.46E-05**	−42.0	**5.59E-04**	89.7	**1.38E-04**
Triacylglycerol	368	170.5	**2.46E-05**	−37.2	**2.07E-03**	69.8	**8.14E-04**

a Mean lipid concentration of healthy control group.

b Mean percentage difference, taking control as reference.

c Significance determined by *t*-test, bold type indicates *P* values were significant after correction for multiple comparisons by the method of Benjamini-Hochberg.

d Mean percentage difference, taking MetS pretreatment (D0) as reference.

e Mean percentage difference, taking MetS control as reference.

At 180 days of pitavastatin treatment, significant reductions in the baseline elevated plasma levels of cholesteryl esters, diacyl- and triacylglycerols, phosphatidylglycerol, phosphatidylethanolamine, and lysophosphatidylcholine toward concentrations in the control group were observed (−23, −42, −37, −36, −22, and −19%, respectively, *P* < 0.05 for all). Clearly, elevated levels of the major lipid classes observed in MetS subjects at pretreatment (D0) were markedly reduced following statin treatment. Plasma concentrations of alkenylphosphatidylcholine and the alkyl- and alkenylphosphatidylethanolamines were not, however, significantly altered (Table 2). Levels of dihydroceramide, which were elevated in MetS as compared with controls, were no longer different to the control group following statin treatment. By contrast, several lipid classes whose levels were lower than controls at baseline were further decreased upon statin therapy, including GM3 gangliosides, alkylphosphatidylcholines, lysophosphatidylcholines, and lysoalkenylphosphatidylcholines. Importantly, these analyses do not reveal the more subtle differences in lipoprotein composition or the changes in the relative concentration of lipid molecular species resulting from pitavastatin treatment.

In order to assess these findings independently of baseline plasma cholesterol levels and changes in those levels, we normalized each lipid class, subclass, and species to nonHDL-C, and then calculated the percent difference between MetS pretreatment (D0) and controls, between posttreatment (D180) and pretreatment (D0) in the MetS group, and between posttreatment (D180) and controls (**Table 3**, supplementary Table 1). These analyses (Table 3) revealed significantly higher diacylglycerol (147%, *P* = 2.1 × 10^−5^) and triacylglycerol (100%, *P* = 3.7 × 10^−5^) relative to nonHDL-C in MetS pretreatment (D0) in CAPITAIN relative to controls, consistent with the hypertriglyceridemia in our MetS subjects. In contrast, most other lipid classes, including the alkyl- and alkenylphospholipids, showed lower levels relative to nonHDL-C in the MetS pretreatment (D0) group compared with the control group. Following statin treatment for 180 days (D180), the decrement in diacyl- and triacylglycerols paralleled that in nonHDL-C. By contrast, significant increments (range 40–60%) in the concentrations of most other lipid classes and subclasses relative to nonHDL-C, and in particular the alkyl- and alkenylphospholipids, were observed (Table 3). Such quantitative increments resulted in a convergence of the lipidomic profile in the MetS group toward that of the control subjects such that of the 20 lipid classes and subclasses that were significantly different between baseline MetS and controls, only five remained significantly different between the posttreatment (D180) and controls (Table 3).

**TABLE 3.** Percent differences between plasma levels of lipid classes (normalized to nonHDL-C) in controls, MetS subjects pretreatment (D0), and MetS post-pitavastatin treatment (D180)

Lipid Class	Relative Concentration (μmol/mmol)*^a^*	MetS Pretreatment (D0) versus Control		MetS Posttreatment (D180) versus MetS Pretreatment (D0)		MetS Posttreatment (D180) versus Control	
		Mean Percent Difference*^b^*	*P^c^*	Mean Percent Difference*^d^*	*P^c^*	Mean Percent Difference*^e^*	*P^c^*
Dihydroceramide	0.24	−12.2	1.22E-01	29.0	**3.52E-02**	13.2	3.53E-01
Ceramide	1.7	−20.7	**1.15E-02**	29.2	**1.21E-03**	2.5	7.57E-01
Monohexocylceramide	2.6	−28.6	**1.39E-02**	8.7	7.27E-02	−22.4	1.02E-01
Dihexosylceramide	2.1	−34.3	**2.10E-03**	17.7	**4.66E-02**	−22.7	7.82E-02
Trihexosylceramide	0.63	−38.2	**9.81E-04**	18.1	**1.95E-02**	−27.1	**1.72E-02**
GM3 ganglioside	1.1	−34.6	**1.66E-04**	20.7	**1.14E-02**	−21.1	**1.72E-02**
Sphingomyelin	113	−25.7	**4.90E-03**	27.3	**4.21E-03**	−5.4	5.35E-01
Phosphatidylcholine	524	−26.6	**4.90E-03**	33.1	**1.21E-03**	−2.3	7.57E-01
Alkylphosphatidylcholine	20.2	−40.3	**3.44E-02**	41.3	**1.42E-03**	−15.6	4.29E-01
Alkenylphosphatidylcholine	12.1	−48.6	**4.90E-03**	39.8	**1.21E-03**	−28.2	1.06E-01
Lysophosphatidylcholine	69.6	−39.8	**1.78E-03**	28.1	**1.14E-02**	−22.9	8.35E-02
Lysoalkylphosphatidylcholine	0.38	−40.7	**1.78E-03**	22.5	**1.71E-02**	−27.3	6.07E-02
Phosphatidylethanolamine	9.1	−4.0	8.02E-01	20.3	5.61E-02	15.4	4.29E-01
Alkylphosphatidylethanolamine	1.1	−51.1	**1.39E-02**	60.0	**4.24E-02**	−21.8	3.92E-01
Alkenylphosphatidylethanolamine	14.0	−51.3	**1.10E-02**	43.9	**5.26E-03**	−30.0	1.59E-01
Lysophosphatidylethanolamine	5.7	−40.7	**4.90E-03**	38.2	**6.56E-03**	−18.0	2.27E-01
Phosphatidylinositol	31.9	−23.5	**2.21E-03**	12.3	1.56E-01	−14.0	1.41E-01
Lysophosphatidylinositol	1.2	−43.6	**6.48E-03**	50.9	**6.35E-03**	−14.9	3.65E-01
Phosphatidylserine	1.0	−45.8	**1.81E-02**	−20.1	5.30E-01	−56.7	**6.97E-03**
Phosphatidylglycerol	0.041	27.8	6.60E-02	−1.2	8.72E-01	26.3	1.99E-01
Bis(monoacylglycero)phosphate	0.0063	−24.5	6.60E-02	57.1	**6.35E-03**	18.7	3.31E-01
Free cholesterol	304	−19.9	**7.55E-03**	9.0	**1.54E-02**	−12.6	1.06E-01
Cholesteryl ester	391	−4.7	4.76E-01	21.2	**5.26E-03**	15.5	1.02E-01
Diacylglycerol	7.9	147.2	**2.13E-05**	−9.5	2.16E-01	123.7	**8.21E-07**
Triacylglycerol	130	100.0	**3.72E-05**	−2.1	8.62E-01	95.8	**1.86E-04**

a Mean lipid concentration of healthy control group (micromoles per millimole nonHDL-C).

b Mean percentage difference, taking control as reference.

c Significance determined by *t*-test, bold type indicates *P* values were significant after correction for multiple comparisons by the method of Benjamini-Hochberg.

d Mean percentage difference, taking MetS pretreatment (D0) as reference.

e Mean percentage difference, taking MetS control as reference.

At the molecular level, all lipid species within the same class were not affected to the same degree by statin treatment, and species within different classes were also differentially impacted. This finding was evident in the phosphatidylcholine and phosphatidylethanolamine subclasses (supplementary Table 1, **Fig. 1A**). For example, the levels of phosphatidylcholine species containing arachidonic acid (PC 36:4 and PC 38:4) were elevated over 60% (*P* = 1.4 × 10^−3^ and *P* = 3.0 × 10^−3^, respectively) following treatment, while the corresponding phosphatidylethanolamine species showed nonsignificant increases of only 20%. In contrast, the phosphatidylethanolamine species containing docosahexaenoic acid (PE 40:6 and PE 40:7) showed larger elevations (28.7%, *P* = 4.8 × 10^−3^ and 44.0%, *P* = 4.3 × 10^−3^) than the corresponding phosphatidylcholine species (supplementary Table 1, Fig. 1A). These specific effects of pitavastatin treatment on lipid species containing arachidonic acid and docosahexaenoic acid were equally evident in the diacylglycerol species; of the 19 species measured, 13 showed a decrease (relative to nonHDL-C) following treatment while 6 showed an increase. All species to show an increase contained either 20:3, 20:4, 22:5, or 22:6 fatty acids. In contrast, the five species that showed the greatest decrease following treatment all contained at least one 18:2 fatty acid (supplementary Table 1, Fig. 1B).

A similar effect was observed with the triacylglycerol species in which the class itself showed no change (relative to nonHDL-C) following treatment; whereas, the TG 18:1/18:1/20:4 and TG 18:1/18:1/22:6 molecular species showed a 35 and 37% increase, respectively (supplementary Table 1). These observations suggest targeted effects of statin action on biological pathways of both lipid and fatty acid metabolism.

### Impact of statin treatment on the plasma lipidome in mixed dyslipidemia and risk for T2D

**Fig. 2.**
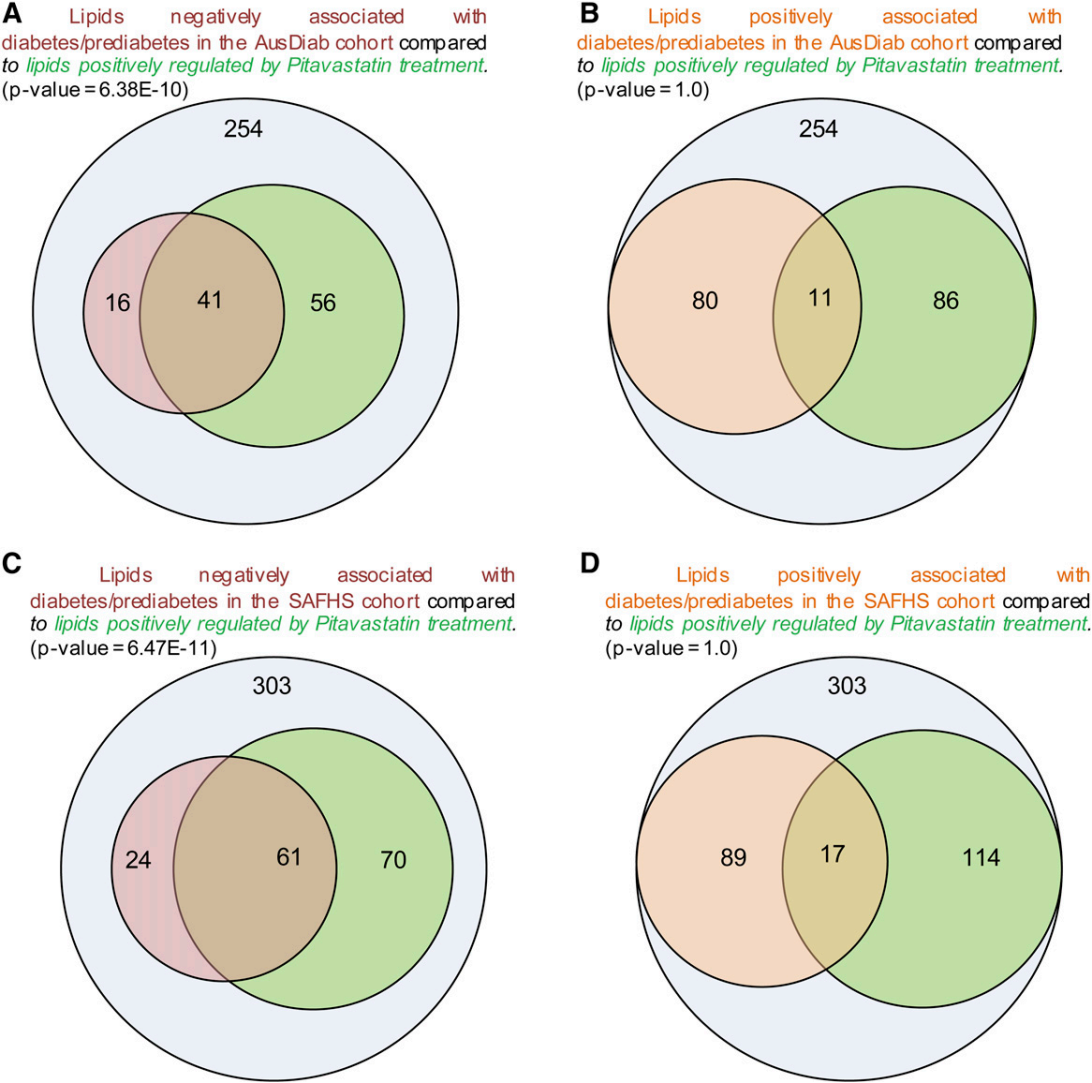
Hypergeometric analysis of the pitavastatin-mediated changes in plasma lipid species in MetS subjects in the CAPITAIN study against plasma lipid species associated with T2D/prediabetes. Plasma lipids in the AusDiab study were normalized to nonHDL-C and the association with T2D/prediabetes determined by logistic regression adjusted for age, sex, BMI, and SBP. Plasma lipids in the CAPITAIN study were normalized to nonHDL-C and the lipid species significantly altered by treatment were determined using a paired Student’s *t*-test. Comparison of these datasets identified 254 common lipid species. Hypergeometric tests were performed to assess the statistical significance of the overlap of the lipid set negatively associated with T2D/prediabetes [n = 57, pink circle (A)] with the lipid set that was upregulated by pitavastatin treatment [n = 97, green circle (A)]. The overlap was significant (*P* = 6.38 × 10^−10^). The intersection of the lipid set positively associated with T2D/prediabetes [n = 91, orange circle (B)] with the lipid set that was upregulated by statin treatment [n = 97, green circle (B)] was not significant (*P* = 1.0). The same analyses in the SAFHS dataset combined with the CAPITAIN dataset resulted in 303 common lipid species. The hypergeometric analyses identified a significant overlap between the lipid set negatively associated with T2D/prediabetes [pink circle (C)] and the lipid set upregulated by statin treatment [green circle (C), *P* = 6.47 × 10^−11^]. The overlap between the lipid set positively associated with T2D/prediabetes [orange circle (D)] and the set upregulated by statin treatment [green circle (D)] was not significant (*P* = 1.0).

In order to assess the potential impact of statin-induced modifications in the plasma lipidome on risk for T2D, we applied a hypergeometric test to assess the relationship between enrichment in lipids associated with T2D on the one hand, and those significantly altered by statin treatment on the other. First, this approach allowed interpretation of those changes in the biological context of lipid metabolic pathways, and second allowed use of this information to generate new hypotheses for validation. To control for the global effect of decreased levels of apoB-containing lipoproteins and the general reduction of plasma lipids resulting from statin treatment, hypergeometric analysis was performed on lipid measurements that had been normalized to nonHDL-C. As described earlier, several lipid classes and species were less abundant at baseline (D0) in the MetS group when normalized to nonHDL-C as compared with the control group; the concentrations of these species increased toward normal relative to nonHDL-C following statin treatment. Thus, we observed a significant over-representation of the lipids that were negatively associated with T2D (following normalization to nonHDL-C) relative to those that were upregulated (i.e., their concentrations increased) by pitavastatin treatment (**Fig. 2A, C**). Similar findings were made when these data were compared with those in both the AusDiab (*P* = 6.38 × 10^−10^) and SAFHS cohorts (*P* = 6.47 × 10^−11^) with 34 of the 41 lipids identified in the AusDiab cohort also identified in the SAFHS. In contrast, lipids that were positively associated with T2D were not significantly enriched in the set of lipids that were positively regulated by statin treatment (Fig. 2B, D). There were only two lipid species that were significantly downregulated in response to pitavastatin treatment (supplementary Table 1).

**Fig. 3.**
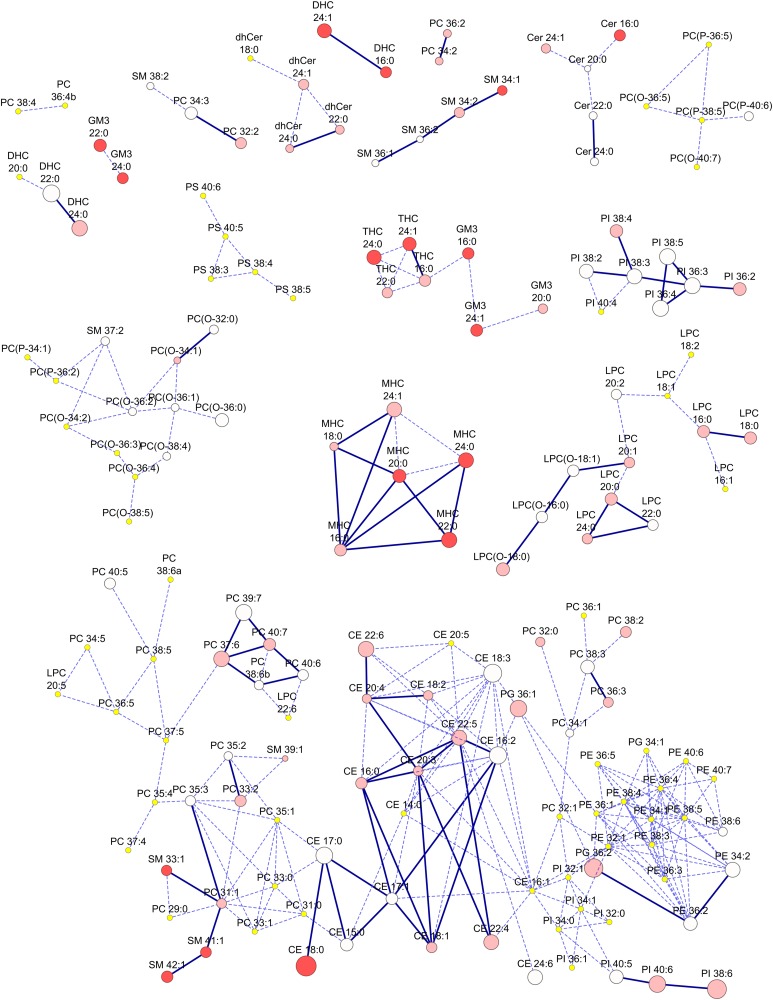
Network analysis of plasma lipids in the AusDiab cohort showing correlated changes between plasma lipids post-pitavastatin treatment. A base network was established as follows: Pearson’s linear correlation was computed between pairs of lipids on all subjects from the AusDiab cohort. Edges (dashed lines) were defined if the correlation between node pairs (lipids) was statistically significant (i.e., Benjamini-Hochberg corrected *P* value ≤0.05). Solid blues edges denote correlated changes between lipids post-pitavastatin treatment. All computed significant correlations were positive. Nodes were sized according to the magnitude of change (difference in mean) effected by statin treatment. Nodes were colored according to the degree of significance of the change (i.e., darker: more significant). Red (or shades of) indicates statistically significant reduction in lipid abundance resulting from statin treatment. Yellow nodes indicate a nonsignificant change in lipid abundance from statin treatment.

Network analysis was then applied to elucidate clusters of lipid species that might be co-regulated, with a view to the biological interpretation of these complex data (**Fig. 3**). The base network identified strong correlations within the lipid classes, in particular those of phosphatidylcholine, lysophosphatidylcholine, phosphatidylethanolamine, phosphatidylinositol, and cholesteryl esters. The sphingolipids presented a more fragmented array with members of the same class often appearing in different clusters. Additional points of correlation between classes were also observed. When we highlighted those correlated modifications between lipids resulting from statin treatment (solid lines), we observed correlated changes in cholesteryl ester, phosphatidylinositol, lysophosphatidylcholine, and monohexosylceramide species with smaller numbers of correlated lipid species in the phosphatidylcholine, sphingomyelin, and other sphingolipid classes.

## DISCUSSION

Our global analysis of statin-induced modification in the abnormal plasma lipid profile in insulin-resistant MetS subjects in the CAPITAIN study has demonstrated a major shift toward that in healthy normolipidemic noninsulin-resistant controls. Indeed, this marked statin-induced shift in circulating levels of multiple molecular lipid species toward levels in the control group suggests that pitavastatin therapy may reduce risk of dysglycemia and T2D associated with the atherogenic mixed dyslipidemia characteristic of insulin-resistant states.

Quantitative and qualitative lipidomic analysis of the impact of statin treatment on our mixed dyslipidemic subjects with MetS who exhibited a prediabetic state at baseline has emphatically demonstrated extensive pleiotropic impact on circulating lipids beyond atherogenic apoB-containing lipoproteins and LDL-C, thereby revealing modulation of lipid levels which are otherwise undetected by standard plasma lipid assays and are potentially clinically relevant. Indeed, significant statin-induced modifications in 18 of 25 plasma lipid classes and subclasses, and in 140 of 330 individual molecular lipid species, were documented. Moreover, mass spectrometry-based lipid profiling has recently identified specific features of the plasma lipidome associated with diabetic and prediabetic states; this signature includes elevated levels of diacylglycerol, phosphatidylethanolamine, and phosphatidylglycerol and decreased concentrations of the alkyl- and alkenyl- species of phosphatidylethanolamine and phosphatidylcholine (16), and was reflected in this study in the MetS participants relative to the healthy controls.

The global shift seen in the abundance of plasma lipid classes and subclasses upon statin treatment may mask differential changes in the lipid composition of individual lipoprotein fractions. To adjust for this effect, we normalized plasma lipid data against nonHDL-C prior to analysis. Consequently, many lipid species show elevation in plasma levels relative to nonHDL-C following pitavastatin treatment, thereby indicating preferential enrichment relative to cholesterol within lipoprotein particles. Applying this normalization to both control and MetS groups highlighted baseline differences in plasma lipid profile between them, and equally emphasized the dramatic shift in multiple lipid classes toward the lipidome of control non-insulin-resistant normolipidemic subjects upon statin treatment. However, while absolute levels of the diacylglycerol and triacylglycerol species were elevated over 2-fold in MetS subjects relative to controls at baseline, they were dramatically reduced in response to statin treatment, such that they were not significantly altered proportionately to changes in nonHDL-C. Similarly, the phosphatidylethanolamine and phosphatidylglycerol classes which, while elevated in the MetS group relative to controls (Table 2), were equally reduced upon statin treatment and so were not significantly altered relative to reduction in nonHDL-C concentrations (Table 3). The metabolism of these lipids may therefore be more tightly linked to the action of the statin than to that of other lipid classes. The network analysis (Fig. 3) identified strong correlations between lipid species within specific classes and, to a lesser extent, correlations between classes most likely relating to common fatty acid precursors. The overlay of correlated lipid levels post-statin treatment was, for the most part, constrained within lipid classes, possibly indicat­ing that pitavastatin exerts a stronger effect at the lipid class level relative to the tissue or plasma fatty acid pools themselves.

Hypergeometric tests facilitated assessment of the significance of the global statin-mediated changes in the plasma lipidome in relation to risk of T2D. Our earlier studies on independent cohorts provided us with a detailed lipid profile associated with T2D (17). The ability of pitavastatin to alter those lipid species preferentially associated with T2D provides a strong argument for an overall neutral or anti-diabetogenic effect, at least with respect to its effect on the metabolism of biologically significant lipids. Following normalization to nonHDL-C, the lipid species that were negatively associated with T2D and positively regulated (normalized) by statin treatment included species of sphingomyelin and glycosphingolipids, a number of species of phosphatidylcholine, and most species of alkyl- and alkenylphosphatidylcholine (Table 3, supplementary Table 2). Not only is plasma sphingomyelin abundance a risk factor for premature atherosclerosis and coronary heart disease (32), but it is also associated with regulation of surface lipid fluidity, and so may impact the functionality of lipoprotein particles. Indeed, both low particle sphingomyelin content and low sphingomyelin/phosphatidylcholine ratio have been positively associated with elevated fractional cholesterol efflux capacity and equally with anti-oxidative activity of HDL particles (25, 33, 34). These findings support the contention that the clinical benefits of statin treatment may go well beyond reduction of atherogenic forms of plasma cholesterol.

The vinyl ether linkage in the alkenyl lipids (plasmalogens) renders them susceptible to oxidation and imparts anti-oxidant properties; in addition, the high proportion of polyunsaturated fatty acids (particularly arachidonic and docosahexaenoic acid) in both the alkyl- and alkenylphospholipids renders them highly susceptible to oxidation. Thus, their negative association with diabetes/prediabetes potentially reflects enhanced oxidative stress under these conditions (17). The statin-induced preferential increment in such lipids relative to nonHDL-C may therefore result from diminished oxidative stress upon treatment. The relative enrichment of phosphatidylcholine and phosphatidylethanolamine species containing the omega 6 and omega 3 polyunsaturated fatty acids is entirely consistent with this postulate (Fig. 1A). However, while we also observed the enrichment of diacylglycerol species containing arachidonic acid (20:4, omega 6) and docosahexaenoic acid (22:6, omega 3) fatty acids, the marked decrease in all diacylglycerol species containing linoleic acid (18:2, omega 6) suggests an upregulation of the biosynthetic pathway from linoleic to arachidonic acid, principally in the liver. The potential role of these fatty acids in cardiovascular event risk, and by association the role of statins in risk reduction, was highlighted recently in the prospective FINRISK study, in which a negative association of omega 6 and omega 3 fatty acids with cardiovascular event risk was documented, and replicated in the Southall and Brent Revisited study (SABRE) and in the British Women’s Health and Heart Study (35). Further to this, recent genome-wide association study analysis combined with functional validation studies has identified the arachidonic acid metabolome as a regulator of cholesterol metabolism, and notably of reverse cholesterol transport (36). In this context, it is relevant that alkenylphospholipids (plasmalogens), in addition to possessing anti-oxidant properties (37), are equally implicated in the regulation of cholesterol esterification and efflux (38) and in endothelial function (39), both of which are highly relevant to atherosclerotic vascular disease. Interestingly, of the 11 lipid species that were positively associated with T2D in the AusDiab study and also upregulated by pitavastatin (Fig. 2C), three were dihydroceramides, while the remainder were species of phosphatidylethanolamine, cholesteryl ester, and di- and triacylglycerol, all of which contained omega 3 or omega 6 polyunsaturated fatty acids. These same species were also positively associated with T2D in the SAFHS. Thus, we should not discount a potential biological role for these lipid species in the interaction between statins and T2D; further studies are clearly required.

In addition to potential effects on oxidative stress, differences in the response of individual species of phosphatidylcholine and phosphatidylethanolamine to statin treatment may also relate to differential effects on the metabolic pathways involved. In this instance, rates of inter-conversion of phosphatidylethanolamine to phosphatidylcholine by sequential methylation catalyzed by phosphatidylethanolamine *N*-methyltransferase may be involved. This metabolic pathway is known to have specificity for longer chain polyunsaturated species of phosphatidylethanolamine (40) and has relevance to both the onset and progression of hepatic steatosis and to VLDL production rates (41). Thus, the direct or indirect action of pitavastatin on the phosphatidylethanolamine *N*-methyltransferase pathway may contribute to its overall effect on the plasma lipidome in prediabetes and insulin resistance, with potential for either a neutral or attenuating effect on risk of development of T2D. Further studies designed to specifically test these hypotheses are required.

### Limitations and future directions

Risk of dysglycemia and T2D associated with statin use is now a complex and controversial issue, given lipid-lowering recommendations in this patient group (42). Large randomized long-term crossover studies involving rigorous comparison of different statins for potential dysglycemic effects in patients at high diabetes risk are required in order to clarify this question. Indeed, limitations in the CAPITAIN study concern the lack of a statin comparator; importantly, comparable data to that presented here for pitavastatin are not yet available for another member of this class in subjects displaying mixed dyslipidemia and insulin resistance. Indeed, we cannot exclude the possibility that the observed effects on the plasma lipidome are not specific to pitavastatin; studies are ongoing to evaluate this hypothesis. The small cohort size, limited to males, was compensated in part by selection of a group of male patients exhibiting a phenotypically consistent mixed dyslipidemia, which included parameters of glucose homeostasis (23). In addition, conditions of blood collection and plasma separation and storage were rigorously controlled in a Clinical Unit, on the one hand, in order to minimize any potential for oxidative lipoprotein modification and, on the other hand, to minimize potential variation in lipid and lipoprotein parameters in relation to diet or physical activity. Given the small cohort size, patients acted as their own controls in this primarily mechanistic investigation; this approach is able to limit confounding effects due to differences in genotypic background and baseline phenotype relative to a placebo group. However, we recognize that regression to the mean in human studies may lead to overestimation of treatment effects in the absence of a placebo control group. Further to these considerations, the high precision of the lipidomic analysis combined with the paired study design provided sufficient power to clearly identify lipid species and classes that were significantly altered in response to statin therapy, even with correction for multiple comparisons. Relating the data to that generated from two large population studies provided further clinical relevance for these findings. These original data now require validation in a long-term prospective study in an independent large cohort of mixed dyslipidemic subjects, preferably to include women, and a statin comparator.

## Supplementary Material

Supplemental Data
