# Supplementary material for: Statin action favors normalization of the plasma lipidome in the atherogenic mixed dyslipidemia of MetS: potential relevance to statin-associated dysglycemia
Source: J Lipid Res. 2015 Dec;56(12):2381–92. doi: 10.1194/jlr.P061143 (PMC4655992; doi:10.1194/jlr.P061143)
Supplement: Supplemental Data [file supp_56_12_2381__index.html]

Statin action favours normalisation of the plasma lipidome in the atherogenic mixed dyslipidemia of metabolic syndrome: Potential relevance to statin-associated dysglycemia — Statin action favors normalization of the plasma lipidome in the atherogenic mixed dyslipidemia of MetS: potential relevance to statin-associated dysglycemia — Supplemental Data 

# Statin action favors normalization of the plasma lipidome in the atherogenic mixed dyslipidemia of MetS: potential relevance to statin-associated dysglycemia

## Supplemental Data

- Supplemental Data (.pdf, 278 KB) - CAPITAIN Exclusion criteria Supplemental Table 1 Supplemental Table 2
