# Supplementary material for: Statin action favors normalization of the plasma lipidome in the atherogenic mixed dyslipidemia of MetS: potential relevance to statin-associated dysglycemia
Source: J Lipid Res. 2015 Dec;56(12):2381–92. doi: 10.1194/jlr.P061143 (PMC4655992; doi:10.1194/jlr.P061143)
Supplement: Supplemental Data [file 10.1194_P061143_jlr.P061143-1.pdf]

SUPPLEMENTAL DATA

Statin action favours normalisation of the plasma lipidome in the atherogenic mixed dyslipidemia of metabolic syndrome: Potential relevance to statin-associated dysglycemia

Peter J. Meikle[1,]*, Gerard Wong*, Ricardo Tan*, Philippe Giral[†] MD, Paul Robillard[†], Alexina Orsoni[†], Neil Hounslow[§], Dianna J. Magliano*, Jonathan Shaw*, Joanne E. Curran**, John Blangero**, Bronwyn A. Kingwell* and M. John Chapman[†].

* Baker IDI Heart and Diabetes Institute, Melbourne, Australia.

[†] Dyslipidemia and Atherosclerosis Research Unit, INSERM UMR-S939, and University of Pierre and Marie Curie, Pitie-Salpetriere University Hospital, Paris, France.

[§] Kowa Research Europe Ltd, Wokingham, United Kingdom.

** South Texas Diabetes and Obesity Institute, University of Texas Health Science Center at San Antonio, Harlingen, TX, USA

[1] **Address for Correspondence**

Peter J Meikle, Baker IDI Heart and Diabetes Institute, 75 Commercial Road, Melbourne VIC 3004

Ph:+613 8532 1770; Fax:+613 8532 1100

Email:peter.meikle@bakeridi.edu.au

Running Title: Effect of Pitavastatin on the plasma lipidome

CAPITAIN Exclusion criteria

All the subjects included in the study did not meet any of the following exclusion criteria:

1. Women.
2. Non-Caucasian.
3. Excessive obesity defined as Body Mass Index (BMI) above 35 kg/m^2, rounded to the nearest whole number.
4. LDL-C > 190 mg/dL at screening.
5. Fasting triglycerides > 400 mg/dL at screening.
6. Diabetes mellitus, defined as a fasting glucose > 7 mmol/L, or taking diabetic therapy at screening.
7. History of symptomatic cardiovascular disease including angina pectoris, acute myocardial infarction, or peripheral arterial disease including intermittent claudication.
8. History of symptomatic cerebrovascular disease, including cerebrovascular haemorrhage, transient ischemic attack, or carotid endarterectomy.
9. A current smoker or have smoked in the preceding 12 months
10. Consume more than 10 g of alcohol (equivalent to one 100 mL glass of table wine) per day
11. Have received statins, fibric acid derivatives, bile acid sequestrants, cholesterol absorption inhibitors (including ezetimibe) or nicotinic acid >500 mg per day in the previous year.
12. Have uncontrolled hypertension (SBP ≥140 mmHg or DBP ≥90 mmHg). Patients may have their hypertension controlled with a calcium channel blocker, but must not receive treatment with a diuretic, beta-blocker, angiotensin converting enzyme (ACE) inhibitor or angiotensin II receptor blocker (ARB). If the patient has previously received treatment with these therapies, they must have been discontinued at least 2 months previously.

13. Any conditions that cause secondary dyslipidaemia or increase the risk of statin therapy including alcoholism, autoimmune disease, nephrotic syndrome, uremia, any viral hepatitis clinically active within 12 months before study entry, obstructive hepatic or biliary disease, dysglobulinaemia or macroglobulinaemia, multiple myeloma, glycogen storage disease, porphyria, and uncontrolled hypothyroidism or hyperthyroidism. Controlled thyroid disease (normal serum thyroid stimulating hormone (TSH) and stable therapy for at least 3 months) is permitted.
14. History of pancreatic injury or pancreatitis, or impaired pancreatic function/injury as indicated by abnormal lipase.
15. Liver injury as indicated by serum transaminase levels (alanine aminotransferase/serum glutamic pyruvic transaminase [ALAT/SGPT], aspartate aminotransaminase/serum glutamic oxaloacetic transaminase [ASAT/SGOT]) >3 x upper limit of the reference range (ULRR).
16. Impaired renal function as indicated by serum creatinine levels >1.5 x ULRR at screening or eGFR by Cockroft formula less than 60 mL/min.
17. History of any muscle disease or unexplained elevation (>3 x ULRR) of serum creatine kinase (CK).
18. Any surgical or medical condition that might significantly alter the absorption, distribution, metabolism or excretion of the study drug, including: history of major gastrointestinal tract surgery *e.g.* gastrectomy, gastroenterostomy or small bowel resection; gastritis or inflammatory bowel disease; current active ulcers, or gastrointestinal or rectal bleeding.
19. Current obstruction of the urinary tract or difficulty in voiding likely to require intervention during the course of the study.
20. Severe acute illness or severe trauma in the preceding 3 months

21. Evidence of symptomatic heart failure (New York Heart Association ([NYHA]) class III or IV): significant heart block or cardiac arrhythmia.
22. History of uncontrolled complex ventricular arrhythmias, uncontrolled atrial fibrillation/flutter or uncontrolled supraventricular tachycardias with a ventricular response rate of > 100 beats per minute at rest. Patients whose electrophysiological instability is controlled with a pacemaker or implantable cardiac device are eligible.
23. History of drug abuse.
24. History of allergy or intolerance to medication (including statins).
25. Current or recent (within 1 week) use of supplements or medications known to alter lipid metabolism including soluble fibre (including > 2 teaspoons Metamucil or psyllium containing supplement per day), or other dietary fibre supplements, fish oils containing Omega 3 oils, 'fat blockers' (*e.g.*, orlistat), or other products at the discretion of the investigator.
26. Any forbidden concomitant medication.
27. Within the exclusion period defined in the National Register for Healthy Volunteers of the French Ministry of Health.
28. Participation to any clinical trial with an investigational drug in the past 3 months preceding study entry
29. Forfeit their freedom by administrative or legal award or who were under guardianship

Supplemental Table 1. Differences between plasma levels of lipid species (normalized to non-HDL-C) in healthy male Control subjects, male MetS subjects pre-treatment (D0) and MetS post-treatment (D180).

		Baseline (D0) vs Control		Follow-up (D180) vs Baseline (D0)		Follow-up (D180) vs Control	
**Lipid species[a]**	**Relative concentration (μmol/mmol)[b]**	**Mean % Difference[c]**	**p-value[d]**	**Mean % Difference[e]**	**p-value[d]**	**Mean % Difference[f]**	**p-value[d]**
dhCer 16:0	0.018	-13.9	7.09E-02	21.5	1.01E-01	4.6	7.43E-01
dhCer 18:0	0.019	3.0	8.43E-01	41.6	8.08E-02	45.8	7.69E-02
dhCer 20:0	0.010	-17.7	5.27E-02	36.5	5.39E-02	12.4	4.87E-01
dhCer 22:0	0.043	-6.7	4.82E-01	27.7	6.89E-02	19.1	2.43E-01
dhCer 24:0	0.10	-22.4	**2.34E-02**	30.5	**4.49E-02**	1.3	9.38E-01
dhCer 24:1	0.045	0.6	9.54E-01	23.4	1.11E-01	24.1	1.39E-01
Cer 16:0	0.098	-20.2	**8.20E-03**	19.8	**1.14E-02**	-4.4	6.46E-01
Cer 20:0	0.035	-13.3	1.91E-01	37.0	**5.41E-03**	18.8	1.37E-01
Cer 22:0	0.24	-11.1	2.27E-01	30.0	**6.74E-03**	15.6	2.23E-01
Cer 24:0	1.0	-27.5	**8.44E-03**	30.2	**5.18E-03**	-5.6	6.55E-01
Cer 24:1	0.35	-8.7	3.89E-01	28.0	**8.08E-03**	16.8	2.25E-01
MHC 16:0	0.37	-28.1	**1.10E-02**	17.3	**2.13E-02**	-15.7	2.08E-01
MHC 18:0	0.050	-27.9	**4.99E-02**	30.0	**1.69E-02**	-6.2	7.56E-01

		Baseline (D0) vs Control		Follow-up (D180) vs Baseline (D0)		Follow-up (D180) vs Control	
**Lipid species**[a]	**Relative concentration (μmol/mmol)**[b]	**Mean % Difference**[c]	**p-value**[d]	**Mean % Difference**[e]	**p-value**[d]	**Mean % Difference**[f]	**p-value**[d]
MHC 20:0	0.064	-27.8	**2.48E-02**	13.7	1.24E-01	-17.8	2.31E-01
MHC 22:0	0.63	-23.4	7.14E-02	5.8	2.72E-01	-19.0	2.04E-01
MHC 24:0	0.86	-31.2	**1.55E-02**	6.0	1.50E-01	-27.1	6.21E-02
MHC 24:1	0.66	-30.4	**1.81E-02**	8.0	3.31E-01	-24.9	7.97E-02
DHC 16:0	1.5	-34.8	**2.97E-03**	21.5	**4.51E-02**	-20.8	9.63E-02
DHC 18:0	0.024	-31.9	**2.07E-02**	30.0	**3.19E-02**	-11.5	4.99E-01
DHC 20:0	0.018	-27.3	5.62E-02	26.5	1.01E-01	-8.0	6.58E-01
DHC 22:0	0.11	-27.3	**3.68E-02**	-0.5	9.76E-01	-27.7	**2.86E-02**
DHC 24:0	0.11	-32.0	**9.00E-03**	3.9	6.94E-01	-29.4	**2.22E-02**
DHC 24:1	0.37	-35.5	**4.74E-03**	11.2	1.94E-01	-28.3	**3.55E-02**
THC 16:0	0.31	-36.3	**1.07E-03**	18.3	**2.77E-02**	-24.6	**2.04E-02**
THC 18:0	0.034	-31.8	**1.29E-02**	37.5	**1.01E-02**	-6.3	7.12E-01
THC 20:0	0.015	-45.6	**5.55E-03**	21.9	2.11E-01	-33.7	6.57E-02
THC 22:0	0.064	-40.4	**3.70E-03**	24.2	7.43E-02	-26.0	7.69E-02
THC 24:0	0.076	-46.2	**1.15E-03**	8.7	2.42E-01	-41.5	**6.33E-03**

		Baseline (D0) vs Control		Follow-up (D180) vs Baseline (D0)		Follow-up (D180) vs Control	
**Lipid species[a]**	**Relative concentration (μmol/mmol)[b]**	**Mean % Difference[c]**	**p-value[d]**	**Mean % Difference[e]**	**p-value[d]**	**Mean % Difference[f]**	**p-value[d]**
THC 24:1	0.13	-37.9	**2.97E-03**	13.3	1.25E-01	-29.7	**2.86E-02**
GM3 16:0	0.23	-35.5	**8.19E-04**	18.3	**2.52E-02**	-23.7	**1.79E-02**
GM3 18:0	0.17	-42.3	**5.18E-04**	38.0	**1.29E-02**	-20.4	6.23E-02
GM3 20:0	0.072	-27.7	**1.17E-03**	24.7	**3.64E-02**	-9.8	2.31E-01
GM3 22:0	0.16	-24.8	**6.29E-03**	14.0	5.94E-02	-14.3	9.91E-02
GM3 24:0	0.18	-41.6	**6.92E-04**	18.9	6.16E-02	-30.6	**9.28E-03**
GM3 24:1	0.25	-32.0	**1.80E-03**	17.9	**3.74E-02**	-19.9	6.43E-02
SM 31:1	0.13	-18.7	1.05E-01	28.6	**3.56E-02**	4.6	7.84E-01
SM 32:0	0.20	-22.9	**2.67E-02**	40.0	6.89E-02	7.9	6.83E-01
SM 32:1	4.7	-22.2	**2.95E-02**	28.3	**1.36E-02**	-0.2	9.89E-01
SM 32:2	0.33	-12.6	1.08E-01	30.2	**1.68E-02**	13.8	2.28E-01
SM 33:1	2.8	-24.4	**3.89E-02**	23.7	**9.45E-03**	-6.4	6.76E-01
SM 34:0	2.0	-30.0	**5.04E-03**	28.0	**1.39E-02**	-10.4	3.46E-01
SM 34:1	48.0	-30.8	**3.06E-03**	24.9	**5.42E-03**	-13.5	1.65E-01
SM 34:2	6.6	-26.0	**2.25E-03**	23.1	**1.48E-02**	-8.9	2.98E-01

		Baseline (D0) vs Control		Follow-up (D180) vs Baseline (D0)		Follow-up (D180) vs Control	
**Lipid species[a]**	**Relative concentration (µmol/mmol)[b]**	**Mean % Difference[c]**	**p-value[d]**	**Mean % Difference[e]**	**p-value[d]**	**Mean % Difference[f]**	**p-value[d]**
SM 34:3	0.051	-26.5	**9.00E-03**	30.3	**2.60E-02**	-4.2	7.56E-01
SM 35:1	1.9	-24.1	5.64E-02	27.8	**1.58E-02**	-3.0	8.64E-01
SM 35:2	0.29	-24.4	**4.67E-02**	30.1	**1.95E-02**	-1.6	9.26E-01
SM 36:1	9.1	-12.4	2.09E-01	34.0	**1.69E-02**	17.4	2.35E-01
SM 36:2	4.3	-19.0	5.60E-02	36.6	**1.29E-02**	10.6	4.87E-01
SM 36:3	0.37	-28.8	**2.37E-02**	30.6	5.12E-02	-7.0	6.83E-01
SM 37:2	0.19	-16.8	2.15E-01	32.0	**3.63E-02**	9.9	6.46E-01
SM 38:1	6.9	-20.8	**3.40E-02**	38.8	**5.41E-03**	9.9	4.17E-01
SM 38:2	2.5	-20.4	**4.98E-02**	34.2	**2.77E-02**	6.8	6.56E-01
SM 39:1	2.3	-21.4	**4.89E-02**	40.7	**5.69E-03**	10.5	5.09E-01
SM 41:1	6.4	-18.8	5.22E-02	20.8	**1.36E-02**	-2.0	8.80E-01
SM 41:2	5.2	-26.8	**1.13E-02**	30.6	**4.64E-03**	-4.3	7.56E-01
SM 42:1	9.2	-28.3	**1.58E-02**	17.1	**3.02E-02**	-16.0	1.87E-01
PC 28:0	0.15	-58.0	9.43E-02	65.1	1.80E-01	-30.6	4.95E-01
PC 29:0	0.031	-21.2	3.15E-01	28.6	3.75E-01	1.3	9.70E-01

		**Baseline (D0) vs Control**		**Follow-up (D180) vs Baseline (D0)**		**Follow-up (D180) vs Control**	
**Lipid species[a]**	**Relative concentration (μmol/mmol)[b]**	**Mean % Difference[c]**	**p-value[d]**	**Mean % Difference[e]**	**p-value[d]**	**Mean % Difference[f]**	**p-value[d]**
PC 30:0	1.3	-24.9	7.32E-02	23.1	2.75E-01	-7.6	7.43E-01
PC 31:0	0.28	-17.8	2.69E-01	29.9	1.97E-01	6.8	7.89E-01
PC 31:1	0.48	-21.1	8.69E-02	25.1	**1.39E-02**	-1.3	9.38E-01
PC 32:0	5.0	-27.9	**3.63E-03**	25.2	**4.26E-03**	-9.7	2.99E-01
PC 32:1	8.5	-11.5	3.28E-01	20.1	1.60E-01	6.3	7.56E-01
PC 32:2	2.4	-23.4	**1.67E-02**	20.9	**4.07E-02**	-7.4	5.37E-01
PC 32:3	0.11	-28.8	**1.04E-02**	28.1	**2.52E-02**	-8.8	4.92E-01
PC 33:0	0.53	-31.3	**2.37E-02**	28.8	**2.13E-02**	-11.5	4.59E-01
PC 33:1	1.3	-14.4	3.34E-01	32.3	6.18E-02	13.3	5.34E-01
PC 33:2	1.1	-14.8	2.28E-01	20.1	1.27E-01	2.4	8.93E-01
PC 33:3	0.025	-29.4	**2.52E-02**	33.7	2.34E-01	-5.7	8.08E-01
PC 34:0	1.3	-30.0	**2.96E-03**	17.9	5.92E-02	-17.5	7.69E-02
PC 34:1	68.4	-25.0	**1.83E-02**	29.7	**5.91E-03**	-2.8	8.27E-01
PC 34:2	104	-29.5	**1.58E-02**	30.1	**1.12E-02**	-8.3	5.31E-01
PC 34:3	5.5	-30.3	**8.20E-03**	14.0	2.88E-01	-20.6	1.28E-01

		Baseline (D0) vs Control		Follow-up (D180) vs Baseline (D0)		Follow-up (D180) vs Control	
**Lipid species[a]**	**Relative concentration (μmol/mmol)[b]**	**Mean % Difference[c]**	**p-value[d]**	**Mean % Difference[e]**	**p-value[d]**	**Mean % Difference[f]**	**p-value[d]**
PC 34:4	0.53	-32.5	**4.15E-02**	42.3	5.39E-02	-4.0	8.63E-01
PC 34:5	0.073	-66.5	**5.67E-03**	62.4	1.50E-01	-45.7	6.82E-02
PC 35:0	0.078	-36.2	**1.95E-02**	25.5	1.04E-01	-19.9	2.35E-01
PC 35:1	2.4	-29.1	**2.38E-02**	32.7	**1.72E-02**	-5.9	7.29E-01
PC 35:2	3.6	-34.3	**1.50E-02**	30.7	**1.36E-02**	-14.1	3.39E-01
PC 35:3	0.48	-22.1	5.63E-02	22.9	7.04E-02	-4.2	7.89E-01
PC 35:4	0.38	1.2	9.35E-01	45.2	**7.93E-03**	46.9	**4.69E-02**
PC 35:5	0.067	-57.3	**1.31E-02**	65.0	**4.60E-02**	-29.5	2.28E-01
PC 36:0	0.096	-33.2	**4.98E-03**	16.0	1.20E-01	-22.5	5.96E-02
PC 36:1	17.4	-28.7	**1.32E-02**	28.6	7.43E-02	-8.3	5.68E-01
PC 36:2	72.1	-31.0	**1.20E-02**	30.5	**1.69E-02**	-9.9	4.59E-01
PC 36:3	48.5	-22.7	**3.49E-02**	23.2	**1.58E-02**	-4.7	7.25E-01
PC 36:4a	7.8	-54.1	**4.55E-03**	16.2	4.58E-01	-46.6	**2.40E-02**
PC 36:4b	43.2	-19.5	8.91E-02	60.6	**1.37E-03**	29.3	7.97E-02
PC 36:5	13.2	-58.9	**3.03E-03**	46.8	7.23E-02	-39.7	**4.85E-02**

		Baseline (D0) vs Control		Follow-up (D180) vs Baseline (D0)		Follow-up (D180) vs Control	
**Lipid species[a]**	**Relative concentration (μmol/mmol)[b]**	**Mean % Difference[c]**	**p-value[d]**	**Mean % Difference[e]**	**p-value[d]**	**Mean % Difference[f]**	**p-value[d]**
PC 36:6	0.28	-40.5	**1.04E-02**	18.9	3.22E-01	-29.3	8.15E-02
PC 37:4	1.9	-28.2	**4.04E-02**	62.0	**4.64E-03**	16.3	4.26E-01
PC 37:5	0.49	-53.6	**7.04E-03**	52.2	**2.37E-02**	-29.4	1.37E-01
PC 37:6	0.18	-3.9	8.60E-01	8.9	4.60E-01	4.7	8.71E-01
PC 38:2	3.5	-34.2	**1.17E-03**	19.3	**3.38E-02**	-21.5	**2.72E-02**
PC 38:3	15.0	2.5	8.70E-01	8.7	5.49E-01	11.4	4.25E-01
PC 38:4	31.8	-17.1	1.40E-01	62.7	**2.97E-03**	34.9	6.13E-02
PC 38:5	20.6	-36.5	**1.04E-03**	38.2	**4.64E-03**	-12.2	2.31E-01
PC 38:6a	2.2	-50.9	**1.24E-03**	37.3	**1.23E-02**	-32.6	**3.08E-02**
PC 38:6b	17.8	-24.1	5.14E-02	28.2	**3.68E-02**	-2.7	8.87E-01
PC 38:7	0.62	-46.6	**1.17E-03**	12.6	2.64E-01	-39.9	**9.28E-03**
PC 39:5	0.27	-40.4	**1.65E-02**	30.7	5.73E-02	-22.1	2.34E-01
PC 39:6	0.62	-32.4	8.52E-02	36.1	**3.38E-02**	-8.0	7.64E-01
PC 39:7	0.020	-48.4	**1.61E-02**	7.8	5.26E-01	-44.4	**4.96E-02**
PC 40:4	0.82	-4.6	7.72E-01	17.1	1.85E-01	11.7	4.59E-01

		Baseline (D0) vs Control		Follow-up (D180) vs Baseline (D0)		Follow-up (D180) vs Control	
**Lipid species[a]**	**Relative concentration (µmol/mmol)[b]**	**Mean % Difference[c]**	**p-value[d]**	**Mean % Difference[e]**	**p-value[d]**	**Mean % Difference[f]**	**p-value[d]**
PC 40:5	6.0	-23.9	**5.81E-03**	19.4	**3.84E-02**	-9.1	2.98E-01
PC 40:6	9.5	-16.0	2.37E-01	22.0	6.16E-02	2.5	8.99E-01
PC 40:7	1.8	-35.0	**1.04E-03**	19.9	6.43E-02	-22.0	6.21E-02
PC 40:8	0.44	-41.5	**3.24E-04**	26.8	**4.07E-02**	-25.8	**2.22E-02**
PC(O-32:0)	0.61	-32.4	**1.77E-02**	27.5	**1.14E-02**	-13.8	3.51E-01
PC(O-32:1)	0.19	-40.9	**1.24E-02**	32.9	**4.26E-03**	-21.4	2.09E-01
PC(O-32:2)	0.035	-55.7	**8.23E-03**	12.7	5.76E-01	-50.1	**2.89E-02**
PC(O-34:1)	1.4	-43.6	**8.20E-03**	36.4	**3.64E-03**	-23.0	1.63E-01
PC(O-34:2)	1.7	-59.0	**2.67E-02**	58.3	**6.58E-03**	-35.2	2.20E-01
PC(O-34:3)	0.047	-67.5	**1.13E-02**	55.1	**3.46E-02**	-49.6	7.69E-02
PC(O-34:4)	0.036	-45.4	**2.95E-02**	25.0	2.35E-01	-31.8	1.63E-01
PC(O-35:4)	0.064	-55.0	**3.19E-02**	63.3	**9.00E-03**	-26.5	3.51E-01
PC(O-36:0)	0.017	-40.4	**5.81E-03**	13.6	3.29E-01	-32.3	**3.55E-02**
PC(O-36:1)	0.14	-47.8	**1.10E-02**	34.4	**1.53E-02**	-29.8	1.26E-01
PC(O-36:2)	0.69	-52.2	**8.78E-03**	33.0	**1.23E-02**	-36.5	7.67E-02

		Baseline (D0) vs Control		Follow-up (D180) vs Baseline (D0)		Follow-up (D180) vs Control	
**Lipid species[a]**	**Relative concentration (μmol/mmol)[b]**	**Mean % Difference[c]**	**p-value[d]**	**Mean % Difference[e]**	**p-value[d]**	**Mean % Difference[f]**	**p-value[d]**
PC(O-36:3)	1.5	-43.7	5.00E-02	42.1	**6.58E-03**	-20.0	4.30E-01
PC(O-36:4)	5.1	-36.2	1.07E-01	46.2	**5.69E-03**	-6.8	8.11E-01
PC(O-36:5)	0.38	-69.0	**1.04E-02**	63.6	1.47E-01	-49.3	8.70E-02
PC(O-38:4)	2.6	-30.5	1.17E-01	34.5	**7.29E-03**	-6.5	7.97E-01
PC(O-38:5)	4.2	-36.9	5.78E-02	42.0	**1.12E-02**	-10.4	6.83E-01
PC(O-40:5)	0.59	-37.7	**6.96E-03**	29.5	**1.67E-02**	-19.3	2.14E-01
PC(O-40:6)	0.37	-39.3	**3.19E-02**	44.0	**1.39E-02**	-12.6	5.87E-01
PC(O-40:7)	0.62	-44.9	**1.31E-02**	34.6	**3.59E-02**	-25.8	1.99E-01
PC(P-32:0)	0.60	-40.4	**5.81E-03**	28.5	**1.41E-02**	-23.4	9.63E-02
PC(P-32:1)	0.097	-52.9	**2.04E-03**	43.8	**4.26E-03**	-32.3	**4.96E-02**
PC(P-34:1)	1.0	-53.7	**1.80E-03**	46.3	**3.01E-03**	-32.3	**4.63E-02**
PC(P-34:2)	2.2	-57.9	**2.96E-03**	53.1	**4.26E-03**	-35.6	5.75E-02
PC(P-34:3)	0.053	-66.9	**1.17E-03**	57.1	**9.12E-03**	-48.0	**1.80E-02**
PC(P-36:2)	0.74	-60.0	**5.67E-03**	34.2	**1.36E-02**	-46.3	**3.67E-02**
PC(P-36:4)	3.5	-39.8	**2.80E-02**	39.4	**6.74E-03**	-16.0	4.30E-01

		Baseline (D0) vs Control		Follow-up (D180) vs Baseline (D0)		Follow-up (D180) vs Control	
**Lipid species[a]**	**Relative concentration (μmol/mmol)[b]**	**Mean % Difference[c]**	**p-value[d]**	**Mean % Difference[e]**	**p-value[d]**	**Mean % Difference[f]**	**p-value[d]**
PC(P-36:5)	0.34	-68.0	**5.81E-03**	51.3	1.52E-01	-51.6	**4.96E-02**
PC(P-38:4)	1.2	-43.5	**4.13E-02**	27.4	**1.69E-02**	-28.0	2.35E-01
PC(P-38:5)	1.8	-47.4	**1.02E-02**	39.6	**8.17E-03**	-26.5	1.65E-01
PC(P-38:6)	0.35	-49.4	**6.29E-03**	35.1	**2.18E-02**	-31.6	8.70E-02
PC(P-40:6)	0.23	-58.5	**5.54E-03**	30.1	**4.01E-02**	-46.1	**3.68E-02**
LPC 14:0	0.57	-27.5	9.01E-02	35.8	1.17E-01	-1.6	9.46E-01
LPC 15:0	0.31	-15.6	2.47E-01	24.9	7.93E-02	5.4	7.97E-01
LPC 16:0	29.0	-30.4	**2.44E-03**	18.1	**4.98E-02**	-17.8	7.97E-02
LPC 16:1	1.2	-29.0	**1.58E-02**	20.4	1.47E-01	-14.5	3.68E-01
LPC 17:0	0.73	-38.3	**5.92E-03**	20.7	**4.52E-02**	-25.6	7.76E-02
LPC 17:1	0.17	-39.1	**7.98E-03**	31.9	**4.07E-02**	-19.6	2.20E-01
LPC 18:0	9.4	-34.5	**2.04E-03**	18.7	1.06E-01	-22.2	6.21E-02
LPC 18:1	8.6	-49.3	**3.05E-03**	41.9	**1.58E-02**	-28.1	7.97E-02
LPC 18:2	12.8	-56.4	**8.63E-03**	49.9	**1.69E-02**	-34.7	1.07E-01
LPC 18:3	0.31	-57.0	**3.90E-03**	55.6	1.11E-01	-33.1	1.05E-01

		Baseline (D0) vs Control		Follow-up (D180) vs Baseline (D0)		Follow-up (D180) vs Control	
**Lipid species[a]**	**Relative concentration (μmol/mmol)[b]**	**Mean % Difference[c]**	**p-value[d]**	**Mean % Difference[e]**	**p-value[d]**	**Mean % Difference[f]**	**p-value[d]**
LPC 20:0	0.060	-53.0	**1.24E-03**	16.9	1.67E-01	-45.1	**9.28E-03**
LPC 20:1	0.11	-50.3	**1.72E-03**	25.0	**3.92E-02**	-37.9	**2.05E-02**
LPC 20:2	0.14	-52.1	**3.92E-03**	23.7	1.07E-01	-40.7	**2.89E-02**
LPC 20:3	1.3	-41.0	**9.48E-03**	32.5	5.22E-02	-21.9	1.81E-01
LPC 20:4	3.0	-43.2	**1.52E-02**	64.1	**3.64E-03**	-6.8	7.56E-01
LPC 20:5	0.63	-72.1	**2.04E-03**	72.3	**2.13E-02**	-51.9	**2.40E-02**
LPC 22:0	0.013	-45.8	**1.17E-03**	22.0	1.07E-01	-33.8	**2.04E-02**
LPC 22:1	0.0090	-42.4	**4.55E-03**	29.3	1.01E-01	-25.6	1.08E-01
LPC 22:5	0.34	-54.4	**1.17E-03**	39.0	**1.36E-02**	-36.7	**2.22E-02**
LPC 22:6	0.90	-51.4	**6.36E-03**	42.0	**4.60E-02**	-31.0	1.16E-01
LPC 24:0	0.033	-49.1	**6.79E-04**	22.7	**2.13E-02**	-37.6	**6.33E-03**
LPC(O-16:0)	0.13	-38.1	**6.83E-03**	22.9	**4.12E-02**	-23.9	1.07E-01
LPC(O-18:0)	0.040	-39.1	**8.99E-03**	17.2	9.06E-02	-28.7	7.93E-02
LPC(O-18:1)	0.092	-41.9	**1.61E-03**	23.4	5.73E-02	-28.3	**4.63E-02**
LPC(O-20:0)	0.0084	-42.2	**5.81E-03**	22.4	7.25E-02	-29.3	7.97E-02

		Baseline (D0) vs Control		Follow-up (D180) vs Baseline (D0)		Follow-up (D180) vs Control	
**Lipid species[a]**	**Relative concentration (μmol/mmol)[b]**	**Mean % Difference[c]**	**p-value[d]**	**Mean % Difference[e]**	**p-value[d]**	**Mean % Difference[f]**	**p-value[d]**
LPC(O-22:0)	0.015	-38.9	**4.57E-03**	28.0	**5.41E-03**	-21.8	1.07E-01
LPC(O-22:1)	0.011	-47.3	**1.24E-03**	22.4	1.06E-01	-35.4	**3.04E-02**
LPC(O-24:0)	0.031	-41.1	**2.04E-03**	25.7	**7.95E-03**	-25.9	**4.69E-02**
LPC(O-24:1)	0.036	-45.6	**1.04E-03**	21.8	**4.22E-02**	-33.8	**1.88E-02**
LPC(O-24:2)	0.0055	-49.1	**2.69E-03**	6.8	5.53E-01	-45.6	**1.06E-02**
PE 32:0	0.0091	-23.8	5.71E-02	31.2	**2.73E-02**	-0.1	9.96E-01
PE 32:1	0.020	6.8	8.04E-01	28.1	5.77E-01	36.7	5.47E-01
PE 34:1	0.33	-0.9	9.60E-01	25.4	1.67E-01	24.3	3.68E-01
PE 34:2	0.59	-3.4	8.68E-01	3.9	8.37E-01	0.4	9.89E-01
PE 34:3	0.035	-16.3	4.63E-01	17.6	6.43E-01	-1.6	9.70E-01
PE 35:1	0.030	-16.6	3.82E-01	32.3	1.45E-01	10.4	7.29E-01
PE 35:2	0.034	-8.6	6.63E-01	11.7	5.15E-01	2.2	9.38E-01
PE 36:1	0.33	-9.1	6.63E-01	32.2	1.95E-01	20.2	4.87E-01
PE 36:2	1.6	-4.6	8.12E-01	10.2	4.98E-01	5.1	8.38E-01
PE 36:3	0.50	-22.1	2.29E-01	15.6	3.17E-01	-10.0	6.83E-01

		Baseline (D0) vs Control		Follow-up (D180) vs Baseline (D0)		Follow-up (D180) vs Control	
Lipid species[a]	Relative concentration (μmol/mmol)[b]	Mean % Difference[c]	p-value[d]	Mean % Difference[e]	p-value[d]	Mean % Difference[f]	p-value[d]
PE 36:4	0.75	-13.9	4.93E-01	22.4	5.71E-02	5.4	8.38E-01
PE 36:5	0.068	-43.2	**1.52E-02**	41.8	2.78E-01	-19.5	4.26E-01
PE 38:3	0.16	31.8	2.17E-01	3.1	9.01E-01	35.9	2.31E-01
PE 38:4	2.4	8.8	7.35E-01	20.4	2.91E-01	31.0	3.51E-01
PE 38:5	0.73	-26.2	1.46E-01	35.8	**9.12E-03**	0.3	9.89E-01
PE 38:6	1.0	-11.3	6.01E-01	29.1	**1.20E-02**	14.5	6.37E-01
PE 40:5	0.026	49.7	1.19E-01	21.2	7.43E-02	81.5	**2.86E-02**
PE 40:6	0.43	17.0	4.40E-01	28.7	**4.78E-03**	50.6	7.69E-02
PE 40:7	0.081	-29.0	1.72E-01	44.0	**4.26E-03**	2.3	9.38E-01
PE(O-34:1)	0.050	-61.0	**1.32E-02**	73.7	**1.37E-03**	-32.2	1.95E-01
PE(O-34:2)	0.031	-60.1	**1.75E-02**	65.5	7.00E-02	-33.9	2.35E-01
PE(O-36:2)	0.026	-62.7	**1.22E-02**	46.4	**1.39E-02**	-45.4	7.69E-02
PE(O-36:3)	0.054	-54.2	**4.31E-02**	60.8	1.27E-01	-26.4	4.57E-01
PE(O-36:4)	0.24	-49.5	**4.21E-02**	74.2	1.25E-01	-12.0	7.70E-01
PE(O-36:5)	0.036	-72.2	**9.78E-03**	55.5	1.06E-01	-56.8	5.95E-02

		Baseline (D0) vs Control		Follow-up (D180) vs Baseline (D0)		Follow-up (D180) vs Control	
**Lipid species[a]**	**Relative concentration (μmol/mmol)[b]**	**Mean % Difference[c]**	**p-value[d]**	**Mean % Difference[e]**	**p-value[d]**	**Mean % Difference[f]**	**p-value[d]**
PE(O-38:4)	0.17	-41.4	**3.73E-02**	50.8	1.53E-01	-11.7	7.44E-01
PE(O-38:5)	0.30	-53.6	**1.81E-02**	64.5	**4.47E-02**	-23.6	3.92E-01
PE(O-40:5)	0.049	-53.3	**8.07E-03**	49.4	**2.97E-03**	-30.3	1.18E-01
PE(O-40:6)	0.024	-14.0	5.64E-01	42.5	**4.26E-03**	22.6	4.36E-01
PE(O-40:7)	0.075	-52.4	**1.46E-02**	31.5	1.64E-01	-37.4	1.05E-01
PE(P-34:1)	0.22	-56.5	**5.04E-03**	66.6	**2.97E-03**	-27.5	1.39E-01
PE(P-34:2)	0.41	-57.6	**2.48E-02**	50.0	**1.58E-02**	-36.4	1.89E-01
PE(P-36:1)	0.53	-58.7	**5.24E-03**	51.2	**1.67E-02**	-37.5	7.69E-02
PE(P-36:2)	0.97	-63.2	**1.26E-02**	48.7	**1.58E-02**	-45.3	7.97E-02
PE(P-36:4)	1.3	-38.4	8.61E-02	48.6	**2.08E-02**	-8.5	7.74E-01
PE(P-38:4)	2.6	-49.8	**3.79E-02**	40.5	**4.98E-02**	-29.5	2.77E-01
PE(P-38:5)	4.3	-52.8	**1.48E-02**	46.3	**8.84E-03**	-31.0	1.72E-01
PE(P-38:6)	1.1	-47.1	**1.58E-02**	39.8	**1.14E-02**	-26.0	2.20E-01
PE(P-40:5)	1.2	-50.0	**4.04E-03**	34.4	**1.58E-02**	-32.8	6.21E-02
PE(P-40:6)	0.79	-54.0	**1.01E-02**	36.6	**1.11E-02**	-37.2	8.15E-02

		Baseline (D0) vs Control		Follow-up (D180) vs Baseline (D0)		Follow-up (D180) vs Control	
**Lipid species[a]**	**Relative concentration (µmol/mmol)[b]**	**Mean % Difference[c]**	**p-value[d]**	**Mean % Difference[e]**	**p-value[d]**	**Mean % Difference[f]**	**p-value[d]**
LPE 16:0	0.73	-35.7	**3.59E-03**	18.9	1.41E-01	-23.6	7.38E-02
LPE 18:0	1.1	-33.7	**4.86E-03**	16.9	1.20E-01	-22.5	7.69E-02
LPE 18:1	1.1	-51.2	**2.67E-02**	59.9	**3.02E-02**	-21.9	4.12E-01
LPE 18:2	1.4	-45.2	**2.95E-02**	31.7	8.74E-02	-27.8	2.35E-01
LPE 20:4	0.72	-34.8	**2.51E-02**	60.8	**2.97E-03**	4.9	8.07E-01
LPE 22:6	0.74	-37.9	**1.79E-03**	54.5	**3.64E-03**	-4.0	7.74E-01
PI 32:0	0.19	-25.9	9.33E-02	-16.4	5.47E-01	-38.1	7.93E-02
PI 32:1	0.28	-20.5	2.52E-01	0.8	9.93E-01	-19.9	6.27E-01
PI 34:0	0.061	-41.5	**4.89E-03**	-3.5	8.96E-01	-43.6	**1.87E-02**
PI 34:1	2.9	-36.5	**3.58E-03**	11.9	5.77E-01	-28.9	7.97E-02
PI 36:1	2.4	-50.7	**1.24E-03**	43.3	5.73E-02	-29.4	6.85E-02
PI 36:2	6.6	-45.4	**2.42E-03**	11.4	3.02E-01	-39.1	**1.49E-02**
PI 36:3	1.7	-32.1	**2.01E-02**	-0.3	9.93E-01	-32.2	7.69E-02
PI 36:4	1.8	-2.6	8.60E-01	0.9	9.72E-01	-1.7	9.38E-01
PI 38:2	0.35	-10.1	3.73E-01	9.6	4.00E-01	-1.5	9.19E-01

		Baseline (D0) vs Control		Follow-up (D180) vs Baseline (D0)		Follow-up (D180) vs Control	
Lipid species[a]	Relative concentration (μmol/mmol)[b]	Mean % Difference[c]	p-value[d]	Mean % Difference[e]	p-value[d]	Mean % Difference[f]	p-value[d]
PI 38:3	2.0	-0.8	9.44E-01	5.6	6.94E-01	4.7	7.65E-01
PI 38:4	10.8	-4.8	4.99E-01	16.3	**4.12E-02**	10.7	3.00E-01
PI 38:5	1.1	-32.0	**7.20E-04**	2.5	8.70E-01	-30.3	**1.43E-02**
PI 38:6	0.34	-32.4	**1.31E-02**	-8.3	5.12E-01	-38.0	**1.93E-02**
PI 40:4	0.14	-7.8	4.53E-01	15.2	3.29E-01	6.2	6.83E-01
PI 40:5	0.58	-36.7	**1.27E-03**	9.8	3.34E-01	-30.5	**1.73E-02**
PI 40:6	0.63	-34.3	**2.37E-02**	-0.1	9.93E-01	-34.4	5.50E-02
LPI 18:0	0.33	-46.2	**1.24E-04**	43.4	**3.57E-03**	-22.8	**2.80E-02**
LPI 18:1	0.29	-57.0	**1.55E-02**	77.7	**3.02E-02**	-23.6	3.68E-01
LPI 18:2	0.29	-48.6	**3.19E-02**	31.5	1.07E-01	-32.4	1.94E-01
LPI 20:4	0.30	-22.7	1.14E-01	54.7	**2.12E-02**	19.6	3.63E-01
PS 36:1	0.34	-45.9	**3.38E-02**	-20.2	6.02E-01	-56.8	**1.09E-02**
PS 38:3	0.10	-40.1	**3.61E-02**	-21.5	5.53E-01	-53.0	**1.06E-02**
PS 38:4	0.43	-46.4	**3.13E-02**	-22.8	5.49E-01	-58.6	**1.76E-02**
PS 38:5	0.031	-40.0	**3.52E-02**	-13.9	2.91E-01	-48.3	**4.13E-03**

		Baseline (D0) vs Control		Follow-up (D180) vs Baseline (D0)		Follow-up (D180) vs Control	
**Lipid species[a]**	**Relative concentration (μmol/mmol)[b]**	**Mean % Difference[c]**	**p-value[d]**	**Mean % Difference[e]**	**p-value[d]**	**Mean % Difference[f]**	**p-value[d]**
PS 40:5	0.043	-39.9	**3.49E-02**	-11.7	6.68E-01	-46.9	**1.68E-02**
PS 40:6	0.060	-52.0	**2.16E-02**	16.9	5.53E-01	-44.0	7.38E-02
PG 34:1	0.0069	1.7	9.20E-01	6.8	8.07E-01	8.6	7.81E-01
PG 36:1	0.019	43.9	**2.95E-02**	0.0	9.99E-01	43.9	6.21E-02
PG 36:2	0.015	19.3	2.60E-01	-6.1	6.95E-01	12.0	6.56E-01
BMP 18:1/18:1	0.0063	-24.5	7.70E-02	57.1	**1.23E-02**	18.7	3.39E-01
CE 14:0	6.3	13.4	4.00E-01	26.8	2.22E-01	-12.6	1.07E-01
CE 15:0	5.7	9.8	4.63E-01	16.4	2.95E-01	43.7	6.23E-02
CE 16:0	111.5	-4.7	5.19E-01	19.6	**4.63E-03**	27.8	1.24E-01
CE 16:1	26.2	2.7	8.70E-01	20.5	2.13E-01	14.0	1.26E-01
CE 16:2	0.85	-6.5	4.91E-01	-2.7	8.70E-01	23.8	2.93E-01
CE 17:0	2.7	-7.3	6.11E-01	2.8	8.38E-01	-9.0	4.30E-01
CE 17:1	9.0	-6.1	4.93E-01	18.0	**2.07E-02**	-4.6	7.86E-01
CE 18:0	6.0	3.9	6.89E-01	-9.4	1.19E-01	10.9	3.08E-01
CE 18:1	68.5	-9.7	2.70E-01	23.4	**3.02E-02**	-5.8	6.38E-01

		Baseline (D0) vs Control		Follow-up (D180) vs Baseline (D0)		Follow-up (D180) vs Control	
**Lipid species[a]**	**Relative concentration (μmol/mmol)[b]**	**Mean % Difference[c]**	**p-value[d]**	**Mean % Difference[e]**	**p-value[d]**	**Mean % Difference[f]**	**p-value[d]**
CE 18:2	76.9	-7.6	4.07E-01	26.7	**1.23E-02**	11.4	3.63E-01
CE 18:3	14.5	-3.0	8.62E-01	-4.6	8.54E-01	17.1	1.45E-01
CE 20:1	0.086	-7.3	5.51E-01	-15.3	1.11E-01	-7.5	6.38E-01
CE 20:2	0.28	11.4	3.63E-01	22.1	1.04E-01	-21.5	9.63E-02
CE 20:3	5.1	4.9	6.18E-01	27.1	**8.17E-03**	36.0	**3.52E-02**
CE 20:4	37.2	5.7	5.77E-01	29.0	**6.51E-03**	33.4	**3.25E-02**
CE 20:5	15.4	-29.8	7.74E-02	24.4	3.35E-01	36.5	**2.04E-02**
CE 22:0	0.049	43.5	**1.83E-02**	-37.1	**1.37E-03**	-12.6	6.37E-01
CE 22:1	0.026	36.5	**4.11E-02**	-20.6	1.78E-01	-9.8	5.49E-01
CE 22:4	0.049	-8.1	2.70E-01	7.1	3.25E-01	8.5	6.76E-01
CE 22:5	0.26	-8.3	3.29E-01	6.9	4.91E-01	-1.5	8.87E-01
CE 22:6	4.6	7.1	5.84E-01	7.1	4.68E-01	-1.9	8.81E-01
CE 24:5	0.0031	2.1	8.75E-01	-17.2	1.11E-01	14.7	4.30E-01
CE 24:6	0.0035	-10.0	3.93E-01	8.7	5.26E-01	-15.5	3.01E-01
COH	303.6	-19.9	**1.04E-02**	9.0	**2.57E-02**	-2.1	8.87E-01

		Baseline (D0) vs Control		Follow-up (D180) vs Baseline (D0)		Follow-up (D180) vs Control	
**Lipid species**[a]	**Relative concentration (µmol/mmol)**[b]	**Mean % Difference**[c]	**p-value**[d]	**Mean % Difference**[e]	**p-value**[d]	**Mean % Difference**[f]	**p-value**[d]
DG 14:0/18:1	0.17	182.6	**2.96E-03**	-12.0	6.35E-01	148.7	**1.68E-02**
DG 14:0/18:2	0.061	208.1	**5.24E-03**	-25.9	2.25E-01	128.3	**2.05E-02**
DG 16:0/16:0	0.18	240.6	**1.15E-03**	-14.4	5.07E-01	191.5	**9.28E-03**
DG 16:0/18:1	1.3	170.5	**6.33E-05**	-10.0	3.87E-01	143.5	**1.20E-04**
DG 16:0/18:2	0.53	186.8	**4.24E-04**	-21.3	**4.92E-02**	125.7	**9.18E-05**
DG 16:0/20:3	0.052	84.2	**1.38E-02**	-4.9	8.04E-01	75.2	**2.80E-02**
DG 16:0/20:4	0.077	166.8	**3.92E-03**	6.1	8.22E-01	183.2	**9.28E-03**
DG 16:0/22:5	0.036	74.3	**2.46E-03**	3.0	8.48E-01	79.6	**6.33E-03**
DG 16:0/22:6	0.055	70.3	**3.57E-02**	1.5	9.52E-01	72.9	1.37E-01
DG 16:1/18:1	0.47	150.0	**2.55E-04**	-13.2	2.87E-01	117.0	**4.79E-03**
DG 18:0/18:1	0.39	158.7	**1.24E-04**	-1.4	9.50E-01	155.0	**5.13E-04**
DG 18:0/18:2	0.15	171.2	**8.66E-04**	-15.3	1.29E-01	129.8	**9.18E-05**
DG 18:0/20:4	0.057	41.8	**2.86E-02**	23.8	**1.89E-02**	75.5	**3.60E-03**
DG 18:1/18:1	2.0	133.5	**6.33E-05**	-5.6	5.72E-01	120.5	**1.64E-04**
DG 18:1/18:2	1.4	155.7	**4.24E-04**	-16.3	1.23E-01	114.1	**4.02E-04**

		Baseline (D0) vs Control		Follow-up (D180) vs Baseline (D0)		Follow-up (D180) vs Control	
**Lipid species[a]**	**Relative concentration (μmol/mmol)[b]**	**Mean % Difference[c]**	**p-value[d]**	**Mean % Difference[e]**	**p-value[d]**	**Mean % Difference[f]**	**p-value[d]**
DG 18:1/18:3	0.21	106.7	**1.80E-03**	-7.0	6.94E-01	92.3	**1.89E-02**
DG 18:1/20:3	0.22	16.2	5.61E-01	13.1	1.97E-01	31.3	3.17E-01
DG 18:1/20:4	0.38	89.3	**9.00E-03**	24.5	1.24E-01	135.7	**2.43E-03**
DG 18:2/18:2	0.19	164.4	**2.76E-03**	-24.7	1.06E-01	99.2	**1.50E-02**
TG 14:0/16:0/18:1	4.2	116.1	**6.40E-03**	-1.6	9.67E-01	112.6	**3.81E-02**
TG 14:0/16:0/18:2	2.2	153.6	**5.55E-03**	-10.2	6.95E-01	127.8	**4.90E-02**
TG 14:0/16:1/18:1	4.4	56.8	1.07E-01	7.7	8.26E-01	68.9	1.87E-01
TG 14:0/16:1/18:2	1.0	78.6	**4.78E-02**	-6.6	8.37E-01	66.9	2.20E-01
TG 14:0/17:0/18:1	1.0	137.6	**9.74E-04**	-0.2	9.97E-01	137.2	**2.43E-02**
TG 14:0/18:0/18:1	0.26	120.6	**1.71E-02**	8.4	8.68E-01	139.2	7.69E-02
TG 14:0/18:2/18:2	0.36	121.2	**7.20E-03**	-21.2	1.97E-01	74.2	**3.29E-02**
TG 14:1/16:0/18:1	0.61	135.6	**1.13E-02**	1.5	9.72E-01	139.2	**4.44E-02**
TG 14:1/16:1/18:0	2.3	128.2	**1.10E-02**	-6.0	8.60E-01	114.5	7.69E-02
TG 14:1/18:0/18:2	0.11	82.0	**8.20E-03**	2.1	9.50E-01	85.9	**2.86E-02**
TG 14:1/18:1/18:1	1.5	103.3	**1.04E-03**	-14.7	2.73E-01	73.5	**1.22E-02**

		Baseline (D0) vs Control		Follow-up (D180) vs Baseline (D0)		Follow-up (D180) vs Control	
**Lipid species[a]**	**Relative concentration (µmol/mmol)[b]**	**Mean % Difference[c]**	**p-value[d]**	**Mean % Difference[e]**	**p-value[d]**	**Mean % Difference[f]**	**p-value[d]**
TG 15:0/16:0/18:1	0.50	149.5	**1.04E-03**	-2.6	9.39E-01	143.0	**1.88E-02**
TG 15:0/18:1/18:1	0.29	107.8	**7.75E-05**	5.6	7.71E-01	119.5	**1.06E-02**
TG 16:0/16:0/16:0	1.1	184.5	**8.44E-03**	-2.4	9.67E-01	177.6	6.21E-02
TG 16:0/16:0/18:0	1.4	140.3	**1.95E-02**	3.9	9.45E-01	149.8	6.82E-02
TG 16:0/16:0/18:1	14.1	143.7	**6.16E-04**	-4.8	8.10E-01	132.0	**5.69E-03**
TG 16:0/16:0/18:2	3.2	166.1	**1.07E-03**	-16.5	1.78E-01	122.3	**3.60E-03**
TG 16:0/16:1/18:1	14.7	96.5	**6.92E-04**	-3.7	8.37E-01	89.3	**9.28E-03**
TG 16:0/17:0/18:0	0.063	82.8	1.41E-01	7.5	8.75E-01	96.4	1.98E-01
TG 16:0/17:0/18:1	0.70	113.3	**2.88E-03**	0.5	9.93E-01	114.3	**1.88E-02**
TG 16:0/17:0/18:2	0.92	111.6	**7.20E-04**	0.9	9.72E-01	113.5	**5.69E-03**
TG 16:0/18:0/18:1	3.1	128.0	**5.92E-03**	8.4	8.37E-01	147.2	**3.64E-02**
TG 16:0/18:1/18:1	28.6	88.2	**6.33E-05**	1.8	8.69E-01	91.5	**2.15E-04**
TG 16:0/18:1/18:2	14.1	97.7	**6.33E-05**	-6.2	4.19E-01	85.6	**1.64E-04**
TG 16:0/18:2/18:2	3.2	106.3	**1.04E-03**	-14.0	1.59E-01	77.5	**6.33E-03**
TG 16:1/16:1/16:1	0.25	119.3	**4.98E-03**	-16.6	3.37E-01	82.9	7.69E-02

		Baseline (D0) vs Control		Follow-up (D180) vs Baseline (D0)		Follow-up (D180) vs Control	
**Lipid species[a]**	**Relative concentration (μmol/mmol)[b]**	**Mean % Difference[c]**	**p-value[d]**	**Mean % Difference[e]**	**p-value[d]**	**Mean % Difference[f]**	**p-value[d]**
TG 16:1/16:1/18:0	0.25	141.0	**1.05E-02**	-4.1	9.10E-01	131.2	**2.80E-02**
TG 16:1/16:1/18:1	1.5	110.5	**1.04E-03**	-17.3	1.11E-01	74.1	**2.44E-02**
TG 16:1/17:0/18:1	1.8	103.3	**8.10E-05**	3.7	8.45E-01	110.9	**6.33E-03**
TG 16:1/18:1/18:1	2.3	59.4	**1.15E-03**	-2.7	8.37E-01	55.0	**1.80E-02**
TG 16:1/18:1/18:2	3.8	73.9	**2.55E-04**	-9.2	3.34E-01	57.9	**1.43E-02**
TG 17:0/18:1/18:1	0.80	77.5	**3.98E-04**	6.6	6.03E-01	89.2	**2.76E-03**
TG 18:0/18:0/18:0	0.026	-10.6	8.73E-01	193.2	4.06E-01	162.2	4.59E-01
TG 18:0/18:0/18:1	0.33	70.6	1.07E-01	66.9	4.98E-01	184.7	2.35E-01
TG 18:0/18:1/18:1	2.4	72.6	**1.81E-02**	31.2	4.58E-01	126.5	7.37E-02
TG 18:0/18:2/18:2	0.39	51.8	**1.95E-02**	2.0	8.70E-01	54.8	**2.40E-02**
TG 18:1/18:1/18:1	5.8	54.8	**2.78E-02**	5.6	7.27E-01	63.4	6.21E-02
TG 18:1/18:1/18:2	2.4	63.2	**1.52E-02**	-8.9	4.57E-01	48.7	7.69E-02
TG 18:1/18:1/20:4	0.73	21.1	1.90E-01	35.5	**2.02E-02**	64.1	**2.80E-02**
TG 18:1/18:1/22:6	0.72	10.3	4.53E-01	37.4	**3.06E-02**	51.6	**4.59E-02**
TG 18:1/18:2/18:2	2.3	68.3	**9.94E-03**	-10.7	3.38E-01	50.3	9.16E-02

		Baseline (D0) vs Control		Follow-up (D180) vs Baseline (D0)		Follow-up (D180) vs Control	
**Lipid species**[a]	**Relative concentration (µmol/mmol)**[b]	**Mean % Difference**[c]	**p-value**[d]	**Mean % Difference**[e]	**p-value**[d]	**Mean % Difference**[f]	**p-value**[d]
TG 18:2/18:2/18:2	0.25	92.1	**2.33E-02**	-28.4	9.63E-02	37.6	3.20E-01
TG 18:2/18:2/20:4	0.19	44.3	**2.67E-02**	7.5	6.36E-01	55.1	7.97E-02

[a] dihydroceramide (dhCer), ceramide (Cer), monohexosylceramide (MHC), dihexosylceramide (DHC), trihexosylcermide (THC), G_{M3} ganglioside (GM3), sphingomyelin (SM), phosphatidylcholine (PC), alkylphosphatidylcholine (PC(O)), alkenylphosphatidylcholine (plasmalogen, PC(P)), lysophosphatidylcholine (LPC), lysoalkylphosphatidylcholine (lysoplatelet activating factor, LPC(O)), phosphatidylethanolamine (PE), phosphatidylinositol (PI), phosphatidylserine (PS), phosphatidylglycerol (PG), cholesteryl ester (CE), free cholesterol (COH), diacylglycerol (DG) and triacylglycerol (TG)

[b] Mean lipid concentration of Healthy Control group (µmol/mmol non-HDL-C).

[c] Mean percentage difference, taking Control as reference.

[d] Significance determined by t-test, p-values were corrected for multiple comparisons by the method of Benjamini-Hochberg.

[e] Mean percentage difference, taking Baseline (D0) as reference.

[f] Mean percentage difference, taking Control as reference.

Supplemental Table 2. Plasma lipid species (normalised to Non-HDL-C) negatively associated with diabetes/prediabetes in the AusDiab or SAFHS cohorts and upregulated by pitavastatin treatment in the Capitain cohort[a].

#	Lipid species negatively associated with diabetes/prediabetes in the AusDiab cohort and upregulated by Pitavastatin treatment[b,c]	Lipids negatively associated with diabetes/prediabetes in the SAFHS cohort and positively regulated by Pitavastatin treatment[b,c]
1	**THC 16:0**	**THC 16:0**
2	**THC 18:0**	**THC 18:0**
3	**GM3 16:0**	**GM3 16:0**
4	**GM3 18:0**	**GM3 18:0**
5	**SM 33:1**	GM3 20:0
6	**SM 34:1**	SM 31:1
7	**SM 34:2**	SM 32:2
8	**SM 36:2**	**SM 33:1**
9	**SM 38:1**	**SM 34:1**
10	**SM 39:1**	**SM 34:2**
11	**SM 41:1**	SM 34:3
12	**PC 31:1**	SM 35:2
13	**PC 33:0**	SM 36:1
14	**PC 34:0**	**SM 36:2**
15	**PC 34:2**	**SM 38:1**
16	**PC 35:1**	SM 38:2

#	Lipid species negatively associated with diabetes/prediabetes in the AusDiab cohort and upregulated by Pitavastatin treatment[b,c]	Lipids negatively associated with diabetes/prediabetes in the SAFHS cohort and positively regulated by Pitavastatin treatment[b,c]
17	**PC 35:2**	**SM 39:1**
18	**PC 35:4**	**SM 41:1**
19	**PC 36:2**	SM 41:2
20	**PC 36:3**	SM 42:1
21	**PC 37:4**	**PC 31:1**
22	**PC 37:5**	PC 32:0
23	**PC 38:2**	PC 32:3
24	**PC 38:5**	**PC 33:0**
25	**PC 38:6a**	**PC 34:0**
26	**PC(O-32:0)**	PC 34:1
27	**PC(O-32:1)**	**PC 34:2**
28	**PC(O-34:1)**	**PC 35:1**
29	**PC(O-34:2)**	**PC 35:2**
30	**PC(O-36:2)**	**PC 36:2**
31	**PC(O-36:3)**	**PC 36:3**
32	**PC(O-36:4)**	PC 36:4b
33	**PC(O-38:5)**	**PC 37:4**
34	**PC(P-32:0)**	**PC 38:2**
35	**PC(P-34:1)**	PC 38:4

#	Lipid species negatively associated with diabetes/prediabetes in the AusDiab cohort and upregulated by Pitavastatin treatment[b,c]	Lipids negatively associated with diabetes/prediabetes in the SAFHS cohort and positively regulated by Pitavastatin treatment[b,c]
36	**PC(P-36:2)**	**PC 38:5**
37	**PC(P-38:5)**	**PC 38:6a**
38	**LPC 18:1**	**PC(O-32:0)**
39	**LPC 18:2**	**PC(O-32:1)**
40	**LPC 20:4**	**PC(O-34:1)**
41	**LPC 24:0**	**PC(O-34:2)**
42		**PC(O-36:2)**
43		**PC(O-36:3)**
44		**PC(O-36:4)**
45		**PC(O-38:5)**
46		**PC(P-32:0)**
47		**PC(P-34:1)**
48		**PC(P-36:2)**
49		**PC(P-38:5)**
50		**LPC 18:1**
51		**LPC 18:2**
52		**LPC 20:4**
53		**LPC 24:0**
54		PC(O-35:4)

#	Lipid species negatively associated with diabetes/prediabetes in the AusDiab cohort and upregulated by Pitavastatin treatment[b,c]	Lipids negatively associated with diabetes/prediabetes in the SAFHS cohort and positively regulated by Pitavastatin treatment[b,c]
55		PC(P-32:1)
56		PC(P-34:2)
57		LPC 20:3
58		LPC 20:5
59		LPC(O-24:0)
60		PE(O-34:1)
61		PE(O-36:2)
62		PE(O-40:5)
63		PE(P-34:1)
64		PE(P-34:2)
65		PE(P-36:2)
66		PE(P-40:5)
67		LPE 18:1
68		LPE 20:4
69		LPE 22:6

[a] Lipid species were normalised to non-HDL cholesterol and a student t-test was used to determine those negatively associated with diabetes/prediabetes in the AusDiab and SAFHS cohorts. The same test was used to define those lipids positively regulated by pitavastatin treatment. Lipids that were both negatively associated with diabetes/prediabetes and positively regulated by pitavastatin are shown.

[b] dihydroceramide (dhCer), ceramide (Cer), monohexosylceramide (MHC), dihexosylceramide (DHC), trihexosylcermide (THC), G_{M3} ganglioside (GM3), sphingomyelin (SM), phosphatidylcholine (PC), alkylphosphatidylcholine (PC(O)),

alkenylphosphatidylcholine (plasmalogen, PC(P)), lysophosphatidylcholine (LPC), lysoalkylphosphatidylcholine (lysoplatelet activating factor, LPC(O)), phosphatidylethanolamine (PE), phosphatidylinositol (PI), phosphatidylserine (PS), phosphatidylglycerol (PG), cholesteryl ester (CE), free cholesterol (COH), diacylglycerol (DG) and triacylglycerol (TG)

[c] Lipids present in both lists are shown bold in a grey background.
